# Closing water productivity gaps to achieve food and water security for a global maize supply

**DOI:** 10.1038/s41598-018-32964-4

**Published:** 2018-10-03

**Authors:** Huifang Zheng, Qianqian Bian, Yulong Yin, Hao Ying, Qinghua Yang, Zhenling Cui

**Affiliations:** 1 College of Agronomy, Henan Agricultural University, Zhengzhou, 45006 China; 2 College of Resources & Environmental Sciences, Key Laboratory of Plant-Soil Interactions, Ministry of Education, China Agricultural University, Beijing, 100193 China

## Abstract

To achieve food and water security, it is as important to close the water productivity (WP) gap (which was defined as the difference between the maximum attainable WP and the currently achieved WP at the field scale) as it is to close yield gaps. However, few studies have provided quantitative estimates of existing WP gaps and constraining factors for global maize production. Using a meta-analysis of 473 published studies covering 31 countries and 5,553 observations (932 site-years), we found the global average WP value for irrigated maize was 18.6 kg ha^−1^ mm^−1^. These WPs varied by factors such as seasonal precipitation, irrigation regimes, soil organic matter and soil pH. In current production systems, there exists a huge scope for improvement in maize WP, but the reported field experiments achieved only 20–46% of potential WP across all countries. Considering the future, raising WP to 85% of potential WP by 2050, a 100% increase in maize production could be achieved with 20% less planted area and 21% less water consumption than in 2005. Closing the WP gap may be critical to ensuring food security and achieving sustainable global agriculture.

## Introduction

Agriculture faces the challenge of ensuring global food security by increasing yield without increasing freshwater consumption^1^. Worldwide, agriculture accounts for around 75% of freshwater withdrawals^2,3^. Global crop water consumption is expected to increase by 41%, from 6,400 km^3^ in 2000 to 9,060 km^3^ in 2050, if no gains in water productivity (WP) are achieved^4^. Maize has a high rate of water consumption, and accounted for 10% of global crop production in the period 1996–2005^5^. Maize production must be increased by 100% (from 2005 levels) to meet the growing demand by 2050^6^. Due to the drought disaster-affected area increasing with global climate change^7^, irrigation is becoming a paramount technology to increase yield for maize production^8,9^. Therefore, improvements in WP in maize production are critical for addressing the dual challenges of global maize supply and water sustainability^1,10^.

The WP has been defined as the grain yield per unit of seasonal water supply, including available soil water in the root zone between sowing and harvest, as well as sowing-to-maturity rainfall and applied irrigation^11^. Many studies have reported the WP of maize production based on field research and modeling studies (or remote sensing)^12–14^. WP studies based on site-specific field measurements are often specific to local climate, soil and management practices^15,16^; few studies have focused on mapping global WP. Modeling studies of WP generally do not account for all constraining factors, and may exclude important variables such as management practices, owing to limited data availability and quality^17,18^. Meta-analysis have been developed to integrate quantitatively-available individual field measurements, and are increasingly used in ecological and biogeochemical studies^19,20^. This approach can be used to create a global map of WP in maize production and its response to various factors (e.g., climate, soil properties and agricultural practices) in different regions.

To achieve food and water security, closing WP gaps is as important as closing yield gaps. However, studies of WP gaps have included only limited information about field measurements of WP. Here, we define the “WP gap” as the difference between the maximum attainable WP and the currently achieved WP at the field scale. WP gaps have been attributed to constraining factors such as unfavorable climate, poor soil quality and improper management^11,21,22^. Many studies have reported WP responses to various constraining factors^23,24^; however, their results relied on site- or region-specific field experiments^17,25^. In addition, there are many potential trade-offs between yield and WP^26–28^. Therefore, there is a growing need for more comprehensive, quantitative analysis of the global factors constraining WP in maize production.

Although many studies reported that improvements in WP had positive benefits, including ensuring food security^1,2^, and reducing water consumption^29^, no detailed global estimates examine the benefits in terms of closing the WP gap in maize production. Given that growing water scarcity, and pressure on increasing demand of global maize supply, it is extremely important to understand how much water is actually consumed in maize production in 2050, as well as how much of water consumption could be saved and how much of maize production could be increased.

Detailed knowledge of the relationships between WP and climate, soil characteristics and management practices is critical to determining how to close WP gaps at the global scale. However, a quantitative synthesis of the response of WP to all constraining factors and an understanding of the exploitable WP gaps at current production levels are still lacking. Here, we conducted a meta-analysis of peer-reviewed studies (5,553 field observations from 473 papers published between 1980 and 2015) to determine WP in different regions with irrigated maize production; evaluate the responses of WP and yield to climate, soil conditions and water management practices; quantify global WP gaps and yield gaps with limited and unlimited water supplies; and illustrate the potential benefits of improving WP for global maize supply and water consumption in the future.

## Results

**Figure 1:**
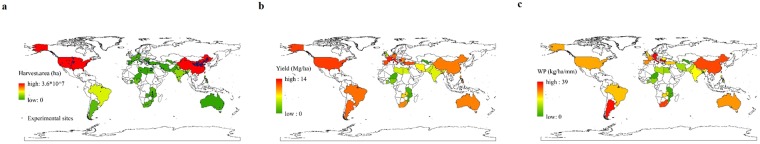
(**a**) Location of study sites in the meta-analysis (*n* = 360 locations) and spatial variation in maize production areas. (**b**) Spatial variation in maize yield based on averages per country (*n* = 31). (**c**) Spatial variation in water productivity based on averages per country (*n* = 31).

We included maize production data from 5,553 field observations data, distributed mainly across 31 countries in Asia, Europe, Africa, North America and South America (Fig. 1).

### Maize yield and WP across regions

Summarizing all 932 site-years and 5,553 field observations, the global mean of irrigated maize grain yield was 9.5 Mg ha^−1^ (Fig. 1b). The global mean of WP in maize production was estimated to be 18.6 kg ha^−1^mm^−1^, and varied from 8.0 to 33.2 kg ha^−1^ mm^−1^ (the 5% and 95% percentiles, respectively) (Fig. 1c). WP was highest in Europe, lowest in Africa, and intermediate in the other regions (Asia, North America and South America) (Table 1). Although maize yield in North America was relatively higher than in other regions, the seasonal water supply was also the highest; thus, WP was relatively low. Although the seasonal water supply in Africa was the lowest, maize yield was also the lowest; thus WP was lower in Africa than in the other four regions. Among 31 countries, those with higher WP were mainly in Europe, including Germany, Bulgaria, and Serbia (38.8, 33.0 and 31.4 kg ha^−1^ mm^−1^, respectively) (Fig. 1c). The lowest WP was observed in African countries: Niger, United Republic of Tanzania, Nigeria and Malawi (5.4, 6.1, 10.3 and 10.4 kg ha^−1^ mm^−1^, respectively) (Fig. 1c).

**Table 1:** The current average maize yield, seasonal water supply, water productivity (WP), WP gap (discrepancy between the highest attained water productivity and actual WP reported for the same ET level) and yield gap (discrepancy between potential yield and actual yield) in Asia, Europe, Africa, North America and South America.

Region	Yield (Mg ha^−1^)	Seasonal water supply (mm)	WP (kg ha^−1^ mm^−1^)	Sample sizes	WP gap (kg ha^−1^ mm^−1^)	Water limited yield (Mg ha^−1^)			Non-water-limited yield (Mg ha^−1^)		
						Current yield (Mg ha^−1^)	Potential yield (Mg ha^−1^)	Yield gap (Mg ha^−1^)	Current yield (Mg ha^−1^)	Potential yield (Mg ha^−1^)	Yield gap (Mg ha^−1^)
Asia	9.08	469	20.5	3206	36	7.71	14.68	6.97	9.65	17.99	8.35
Europe	10.20	464	25.6	182	29	8.72	12.32	3.60	11.25	19.78	8.52
Africa	5.25	436	10.4	435	41	4.74	9.69	4.95	5.82	13.80	7.98
North America	11.15	746	15.9	1509	39	9.39	13.21	3.82	11.64	17.25	5.61
South America	12.08	572	20.9	221	24	12.64	14.44	1.83	12.07	17.5	5.43

### Water supply, management practices and soil properties affect WP and yield

In irrigated maize production, there was no significant correlation between yield (or WP) and seasonal precipitation (Table 2). To further elucidate the relationship between yield (or WP) and seasonal precipitation amount, all data were grouped into three precipitation ranges: <200 mm, 200–400 mm, and >400 mm (Table 2). When seasonal precipitation was <200 mm, both yield and WP increased significantly with increasing precipitation. Conversely, both yield and WP decreased when seasonal precipitation was >400 mm. There was no significant correlation between yield (or WP) and seasonal precipitation when precipitation was in the range of 200–400 mm. Regardless of the amount of precipitation, WP decreased with increasing levels of irrigation. When seasonal precipitation was ≤400 mm, yield increased with increased irrigation, but decreased with increased irrigation when seasonal precipitation was >400 mm (Table 2).

**Table 2:** Correlations between maize yield/water productivity (WP) and precipitation levels, irrigation amount and soil variables.

Variable	Yield			WP		
	n	Corr. C	*p*	n	Corr. C	*p*
Seasonal precipitation (mm)	3274	−0.01	0.584	3274	0.004	0.808
Seasonal precipitation <200 mm	978	0.159	**<0.001**	978	0.286	**<0.001**
Seasonal precipitation 200–400 mm	1551	−0.024	0.346	1551	0.005	0.833
Seasonal precipitation >400 mm	745	−0.082	**0.026**	745	−0.240	**<0.001**
Irrigation amount (mm)	2924	0.143	**<0.001**	2924	−0.400	**<0.001**
Irrigation amount (Seasonal precipitation <200 mm)	906	0.260	**<0.001**	906	−0.433	**<0.001**
Irrigation amount (Seasonal precipitation 200–400 mm)	1426	0.219	**<0.001**	1426	−0.366	**<0.001**
Irrigation amount (Seasonal precipitation >400 mm)	592	−0.081	**0.050**	592	−0.474	**<0.001**
Soil organic matter (g kg^−1^)	1780	0.404	**<0.001**	1780	0.289	**<0.001**
Soil bulk density (g cm^−3^)	2093	−0.115	**<0.001**	2093	−0.137	**<0.001**
Soil pH	2172	−0.174	**<0.001**	2172	0.215	**<0.001**

n, number of datasets included in the correlation analysis; Corr. C., Pearson’s correlation coefficients; *P*, *P*-value of correlation analysis and the values in boldface indicate statistical significance at the *P* < 0.05 probability level.

Soil properties, such as SOM, soil bulk density, and soil pH, played an important role in regulating WP and maize yield. Both yield and WP were significantly positively associated with SOM, suggesting that increased SOM helped to improve maize yield and WP. There were negative relationships between soil bulk density and WP. Maize yield had a strong negative correlation with soil pH; conversely, there was a significant positive correlation between WP and soil pH (Table 2).

**Figure 2:**
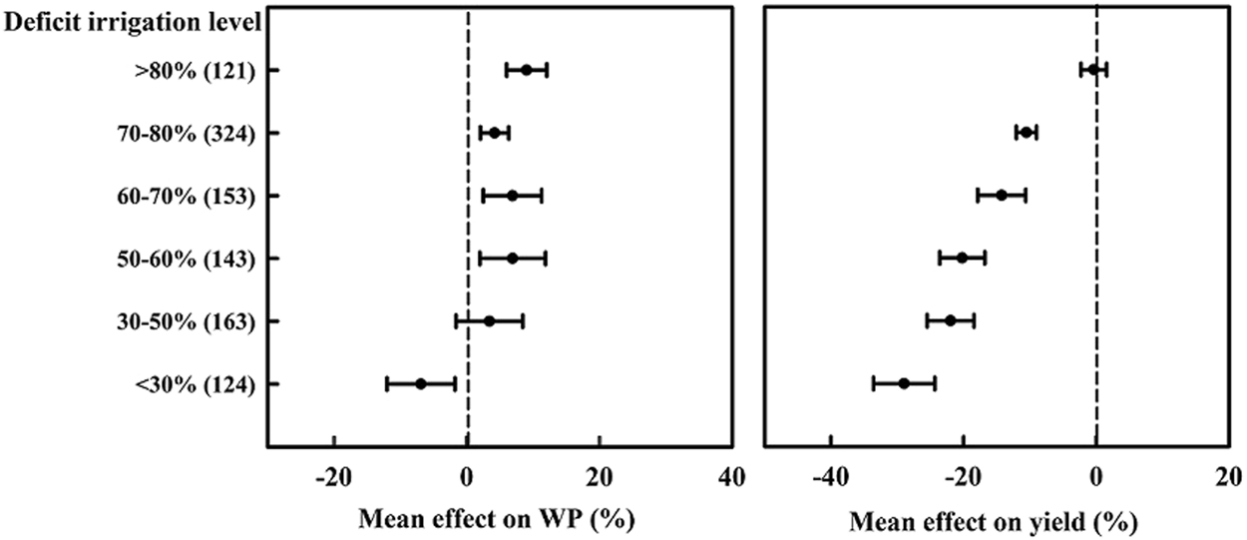
Effects of different deficit irrigation levels on maize yield and water productivity (WP). The number of experimental observations is indicated in parentheses. Confidence intervals that do not overlap between categories indicate significant differences.

Deficit irrigation plays an important role in improving yield and WP in actual production. Here, deficit irrigation is defined as the application of irrigation water in lower amounts than the seasonal irrigation water supply requirements. For example, an 80% deficit in irrigation means that the amount of irrigation water supplied was 20% less than the water requirements. When deficit irrigation was >80%, WP increased by 7.1% with no loss of yield. In areas with 50–80% deficit irrigation, WP increased by 4.2–6.9%, but yield decreased by 10.5–20.2%. WP did not change significantly when 30–50% deficit irrigation was applied, but yield decreased sharply (−22%), compared to 50–60% deficit irrigation. Deficit irrigation levels of <30% reduced both maize yield (−29%) and WP (−6.9%) (Fig. 2).

### Potential WP and WP gap across regions

**Figure 3:**
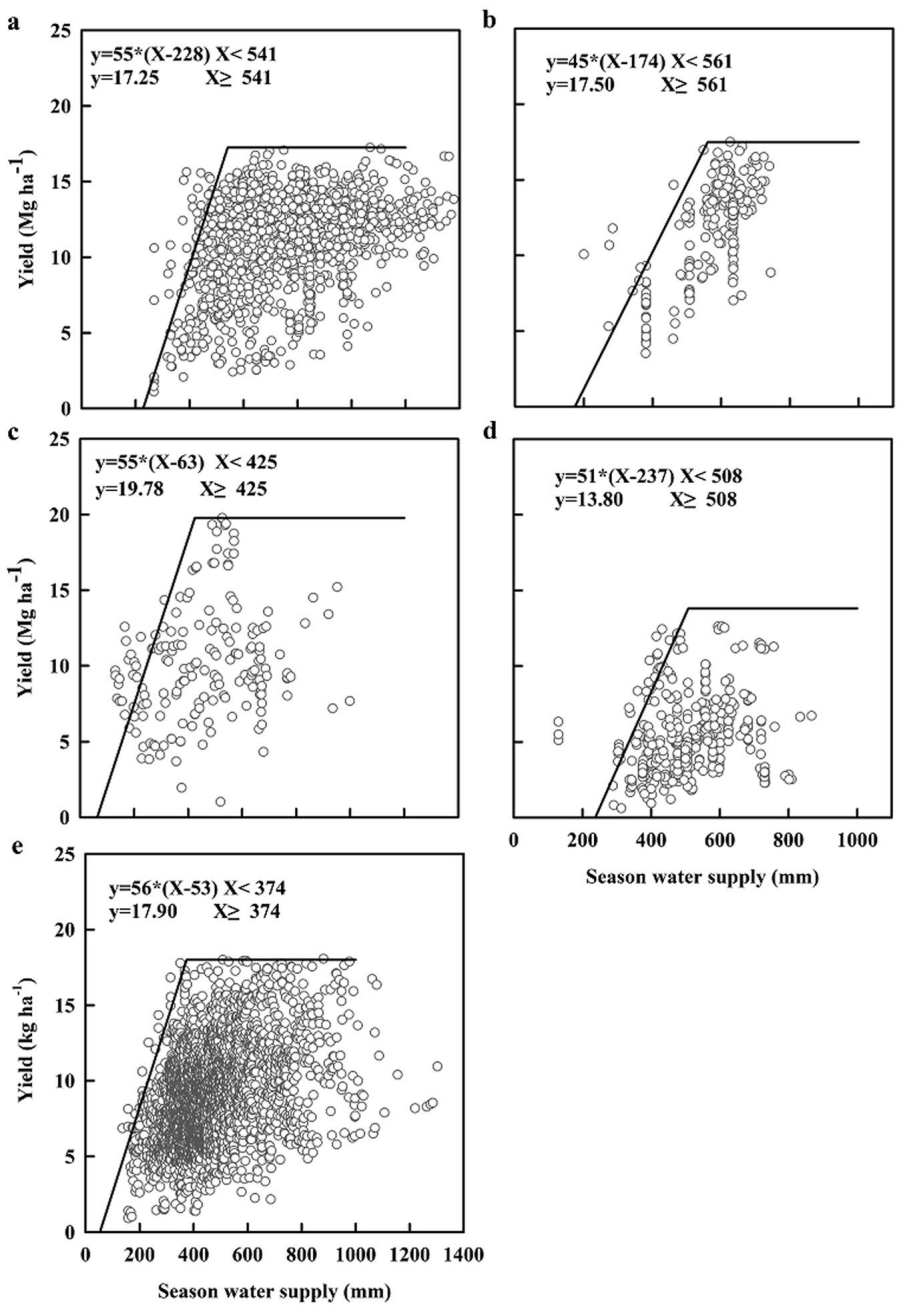
Maize grain yield vs. seasonal water supply measured in experiment fields in (**a**) North America, (**b**) South America, (**c**) Europe, (**d**) Africa, and (**e**) Asia, respectively. The line indicates the WP boundary function.

We established the boundary WP function of maize using all collected data (Fig. 3) using the following equation: Y_p_ = WP_p_ × (seasonal water supply − soil evaporation)^30^, Where: Y_p_ is the potential attainable yield, WP_p_ is the estimated potential WP. Estimated potential WP values were 55, 45, 56, 55, and 51 kg ha^−1^ mm^−1^ for North America, South America, Asia, Europe, and Africa, respectively. The corresponding current mean WP was 15.9, 20.9, 20.5, 25.6, and 10.4 for these five regions, respectively. Calculated WP gaps were in the order Africa (41 kg ha^−1^ mm^−1^) > Asia (36 kg ha^−1^ mm^−1^) > North America (39 kg ha^−1^ mm^−1^) > Europe (29 kg ha^−1^ mm^−1^) > South America (24 kg ha^−1^ mm^−1^). These large WP gaps indicate that the current field experiments achieved only 20–46% of potential WP across all five regions (Table 1).

To further quantify the contribution of water and other factors to yield gaps, we evaluated non-water-limited potential yields gaps in Europe, Asia, North America, South America and Africa based on seasonal water supplies of >425, 374, 541, 561 and 508 mm, respectively (Fig. 3). The greatest non-water-limited potential yield was in Europe (19.8 Mg ha^−1^, *n* = 106), followed by Asia (18.0 Mg ha^−1^, *n* = 2259), South America (17.5 Mg ha^−1^, *n* = 217), North America (17.3 Mg ha^−1^, *n* = 1184) and Africa (13.8 Mg ha^−1^, *n* = 204). The current yields from field experiments were 11.64, 12.07, 9.65, 11.25 and 5.82 Mg ha^−1^ for North America, South America, Asia, Europe, and Africa, respectively. The corresponding yield gaps were 5.61, 5.43, 8.35, 8.52 and 7.98 Mg ha^−1^ for North America, South America, Asia, Europe, and Africa, respectively. Additionally, the water-limited potential yields were 14.68 Mg ha^−1^ in Asia (*n* = 949), followed by South America (14.44 Mg ha^−1^, *n* = 4), North America (13.21 Mg ha^−1^, *n* = 325), Europe (12.32 Mg ha^−1^, *n* = 76) and Africa (9.69 Mg ha^−1^, *n* = 231). Estimated yield gaps were 6.97, 1.83, 3.82, 3.60, and 4.95 Mg ha^−1^; 69%, 69%, 54%, 69% and 46% of potential yields were achieved in North America, South America, Asia, Europe, and Africa, respectively (Table 1).

### Water demand for maize production in 2050

Global maize production in 2005 was used as the reference scenario (S0). We analyzed three main scenarios for the target year 2050: no improvements in WP (S1 scenario); achievement of 50% of potential WP (S2 scenario); and achievement of 85% of potential WP (S3 scenario). Global maize production in 2005 was 0.71 billion tons (BT), with 0.20, 0.09, 0.05, 0.29 and 0.06 BT produced in Asia, Europe, Africa, North America and South America, respectively. Estimated water consumption from both precipitation and irrigation was 392.8 cubic kilometers (S0). Tilman *et al*.^6^, predicted that maize production must be double (from 2005) to meet the growing demand by 2050. Doubling maize production by 2050 will require an increase of 100% in planting area and 100% in water consumption (785.61 cubic kilometers) if no gains in WP are made (S1) (Table 3). Such a large increase in water consumption would have major environmental consequences at local, regional, and global scales, and impose a high environmental cost in terms of groundwater depletion and losses in biodiversity.

**Table 3:** Total water demand and land area required for food security in 2050 under three different scenarios. S1, S2 and S3 represent the current WP, 50% of potential WP and 85% of potential WP, respectively. Global maize production in 2005 is used as the reference scenario (S0).

Scenario	Region	Harvest area (Billion ha)	Total production (Billion Mg)	Total water use (Cubic kilometers)	Increased planting area (Billion ha)
S0	Asia	0.05	0.20	95.51	0
	Europe	0.01	0.09	33.56	0
	Africa	0.03	0.05	48.43	0
	North Americas	0.03	0.29	183.39	0
	South Americas	0.02	0.06	30.98	0
	**Total**	**0.14**	**0.69**	**392.8**	**0**
S1	Asia	0.05	0.40	192.89	0.05
	Europe	0.01	0.17	67.13	0.01
	Africa	0.03	0.10	96.86	0.03
	North Americas	0.03	0.58	366.79	0.03
	South Americas	0.02	0.13	61.95	0.02
	**Total**	**0.14**	**1.38**	**785.61**	**0.14**
S3	Asia	0.05	0.40	141.22	0.02
	Europe	0.01	0.17	62.49	0.01
	Africa	0.03	0.10	39.50	0
	North Americas	0.03	0.58	228.70	0.01
	South Americas	0.02	0.13	57.55	0.01
	**Total**	**0.14**	**1.38**	**529.46**	**0.06**
S3	Asia	0.05	0.40	83.07	(0)
	Europe	0.01	0.17	36.76	0
	Africa	0.03	0.10	23.24	(0)
	North Americas	0.03	0.58	134.53	(0)
	South Americas	0.02	0.13	33.85	0
	**Total**	**0.14**	**1.38**	**311.45**	**(0)**

If current WP increases to 50% of potential WP (S2), total water consumption is expected to be 529.46 cubic kilometers, which would be only 67.4% of that in S1. If current WP increases to 85% of potential WP (S3) (47.6, 46.8, 43.4, 43.4 and 38.3 kg ha^−1^ mm^−1^ in Asia, Europe, Africa, North America and South America, respectively), doubling maize production could be achieved with only 311.45 cubic kilometers water consumed and 20% less planting area required. In this scenario, the water demand would be only 39.6% of the demand in the S1 scenario, and 20.7% lower than the demand in the S0 scenario (Table 3).

## Discussion

### Comparison with other studies

In this study, the global average WP of irrigated maize was 18.6 kg ha^−1^ mm^−1^, which is substantially higher than the results of other studies (e.g., 14.3 kg ha^−1^ mm^−1^ in a study by Liu^18^ that employed the GEPIC model). This may be attributed to the WP, which significantly increased with time^31^. In this study, most of our data (78%) were from after the year 2000, whereas Liu^18^ focused on the year 2000. It was also reported that the average WP increased from 13.5 kg ha^−1^ mm^−1^ in 1980 s to 19.8 kg ha^−1^ mm^−1^ in 2000 s due to the improvements in fertilizers, cultivars and other management practices^32^. The WP in the present study was representative of irrigated agricultural systems, whereas that of Liu^18^ was representative of rain-fed and irrigated systems. Our results are similar to the global average (18 kg ha^−1^ mm^−1^) reported by Zwart and Bastiaanssen (2004)^13^. The latter used a limited dataset with 223 data points from 10 different countries.

### WP variation and the effecting factors

A great deal of variation in WP at the country level was observed, from an average of 5 kg ha^−1^ mm^−1^ in Uzbekistan to an average of 38.75 kg ha^−1^ mm^−1^ in Germany. At the regional scale, WP also varied from 10.3 in Africa to 25.4 kg ha^−1^ mm^−1^ in Europe (Fig. 1, Table 1 and Supplementary Table 1). Many previous studies also found large variations in WP^5,13,14^, owing to differences in climate, site-specific biophysical conditions, agronomic management practices, etc.^5,10^.

In our study, WP values significantly decreased with increasing irrigation amount, regardless of the level of precipitation, indicating that increases in water consumption through irrigation were not offset by increases in maize yield. This may be because the maize was unable to fully utilize seasonally-available water owing to percolation below the root zone or water remaining in the ground at physiological maturity^17,33,34^. Maize grain yields have positively and negatively correlated with irrigation amounts when seasonal precipitation was <400 mm and >400 mm, respectively. Similar results were reported that yield increased by about 80% with supplementary irrigation under semi-arid conditions in southern India^35^. However, supplemental irrigation in high-precipitation regions can decrease yield^25,36^. These results imply that, for irrigated maize, significant improvements in WP and yield can be achieved in low-rainfall regions because supplemental irrigation helps to reduce water stress^37^. Conversely, high levels of precipitation may cause nutrients to be leached from the root zone through percolation and runoff, negatively influencing maize yields and WP^25^.

Deficit irrigation is considered a promising option for improving WP^26,38^. WP increased without any yield losses when deficit irrigation was >80%. However, the favorable and negative impacts of deficit irrigation on yield and WP depend on water supply and deficit irrigation level. For instance, Kirda^28^ reported that grain yield was significantly reduced by 10–25% under 50% deficit irrigation compared with full irrigation. In our study, deficit irrigation at levels below 60% resulted in both lower WP and lower yield. Similar results also indicate that deficit irrigation levels should be relatively high for high yield and WP^38^.

Soil properties significantly affected WP and maize yield. Both yield and WP were positively correlated with SOM. Beneficial effects of increased SOM on yield have also been reported in China^39^ and India^40^. Several studies have reported that certain soil management practices increased SOM and soil water-holding capacity, and promoted rooting and increased crop nutrient and water uptake, ultimately increasing yield and WP^41,42^. In the present study, WP was significantly negatively correlated with soil bulk density (Table 2). In actual production, high soil bulk density is often associated with increased water consumption and reduced yield^43,44^.

### Closing the WP gap for global maize supply and water sustainability

In our study, the estimated potential WP based on the boundary function was 48–56 kg ha^−1^ mm^−1^ in different maize production regions. Similar studies reported maximum attainable WP values of 69 kg ha^−1^ mm^−1^ for global maize production^22^, and 60.5 kg ha^−1^ mm^−1^ under plastic mulching in the Loess Plateau in China^45^. The corresponding WP values based on field measurements were 10.3–25.4 kg ha^−1^ mm^−1^ for different regions of maize production (Table 1). The large WP gaps (29–41 kg ha^−1^ mm^−1^) suggest great potential for improving water productivity to support global food security.

Improvements in WP in maize production are critical for addressing the dual challenges of global maize supply and water sustainability^1,10^. Our results suggest that maize production could be increased by 100% by 2050 with 20% less planting area and 28.9% less water consumption compared with 2005, if farmers worldwide could approach WPs equivalent to 85% of their potentials. Similar studies have reported that improvements in the WP of irrigated cropland could reduce total water consumption by 8–15% in precipitation-limited regions^10^.

Many possibilities exist for large gains in WP to close WP gaps. In actual production, WP improved to 57.1 kg ha^−1^ mm^−1^ by optimum use of the irrigational water in Bulgaria^46^, approaching the potential WP. The combination of water irrigation lateral spacing and partial mulching also approach the potential WP in China^47^. Some caution is required though. Such as, some appropriate strategies for closing WP gaps are region-specific and vary from country to country. Thus, management strategies that aim to increase WP must be tailored to local contexts, depending on factors such as local climate (e.g., rainfall), soil properties, traditional management technologies and economic considerations^1,48^. Further studies on the interacting effects of such factors are required to inform optimal management strategies in different regions. In addition, improvement in both WP and yield may also depend on policies to educate about and award good management practices, aligning the incentives of producers, resource managers and society, and providing a mechanism for dealing with trade-offs^49^.

### Uncertainty of our analysis

Although global data for WP with irrigated maize production was integrated from individual field results across multiple environments and field management practices, some uncertainties exist in this study. First, other field management practices, such as irrigation techniques^50^, nutrient management^42^, varieties and plant populations^24,51^, tillage and soil mulching^12,52,53^, may affect maize WP and yield, although those factors were difficult to investigate in our analysis due to a lack of information from the individual studies. Additionally, while critical soil and environmental factors were considered in our analysis, other experimental variables which may affect maize and yield and WP were not provided in many studies, such as soil water supply, effective accumulated temperature, sunshine hours, soil texture, etc.

Second, estimated WP based on experimental field plots do not accurately represent the WP achieved in farmers’ fields. Generally, WP is greater in well-managed research experiments than when the same practices are applied by farmers in production fields. Thus, future research should focus on collecting measurements from farmers’ fields to generate more robust outcomes. Third, potential WP can vary greatly even within a given region, depending on local soil type and management practices. For example, one study found that potential WPs were 47.5 and 60.5 kg ha^−1^ mm^−1^ under no mulching and mulching conditions, respectively, in the Loess Plateau of China^45^. Thus, more effort are needed in evaluating potential WP under specific conditions or on smaller scales to minimize the current large uncertainties in global or regional estimates.

Fourth, future climate change may affect maize yield and WP^54,55^, although predicting these changes are complicated and general conclusions have not been made due to the intricate interactions of temperature, precipitation and CO_2_. For example, it was reported that maize WP will decline about −2.0% to −36.1% because of high temperatures resulting in evapotranspiration intensification^55^. In contrast, several previous studies reported that climate change may have a positive effect on WP because of increasing CO_2_ concentration^56^ or decrease in precipitation^57^. Fifth, the uneven distribution of sample numbers among countries may affect the credibility of the current WP and yield in our study. Estimated weighted global mean of WP by harvest area was 18.6 kg ha^−1^ mm^−1^, which was similar to the 18.0 kg ha^−1^ mm^−1^ based on observed data in this analysis. Greater attention in the future should be paid to field observations of WP in less-studied regions to minimize data biases.

## Conclusions

Elucidating the nature of WP gaps in global maize production will support the development of strategies to increase agricultural productivity. Our results found that current field experiments achieved only 20–46% of potential WP on a global basis. The maize WP gap could be reduced by improving environmental factors and enhancing agricultural management practices, such as improve SOM and deficit irrigation etc. In the future, closing WP gaps in intensive cropping systems is compatible with increased crop productivity and reduced water consumption, supporting global food and water security, and may be critical to achieving sustainable global agriculture.

## Materials and Methods

### Data collection

We searched the peer-reviewed literatures for publications between 1980 and 2015 that reported on water in maize production using the Web of Science database and the China National Knowledge Infrastructure. Different combinations of searching terms included “maize” or “corn” and “evapotranspiration (ET)” or “seasonal water supply” and “irrigation” and “water use efficiency” or “water productivity” in the abstract or keywords were used for data extraction. We screened the publications based on the following criteria: the data had to be generated from field experiments, rather than pot experiments, experiments under rain-proof shelters or model simulations; the details of experimental sites had to be provided; and maize yield and seasonal water supply had to be documented or calculated based on published data; only irrigated maize were considered. All data were obtained directly from tables or indirectly from graphs using GetData Graph Digitizer^58^. In total, we collected 5,553 field observations from 932 site-years and 473 published papers conducted in 31 countries. Measurements from different sites under the same year or different years under the same sites within a single study were considered as an independent year-site.

These 31 countries accounted for 72% of the global maize harvest area and 81% of global maize production between 2010 and 2016^59^. Similar to previous studies^60,61^, these countries were divided into five global regions: North America, South America, Asia, Europe, and Africa. Combined with our database, Asia included eight countries (China, India, Iran, Lebanon, Pakistan, Bangladesh, the Philippines, and Uzbekistan); Europe covered nine countries, (Bulgaria, France, Germany, Italy, Romania, Serbia, Spain, Turkey); Africa contained Egypt, Ghana, Libya, Malawi, Mozambique, Niger, Nigeria, South Africa, Tanzania, and Zambia; South America two countries (Argentina and Brazil) and North America the United States. Note that the limited sample sizes from Australia (n = 10) was judged insufficient to include in this study, hence Oceania was not represented.

For each literature citation selected, the following original documented information was compiled: maize yield; seasonal water supply; WP; experiment locations (longitude and latitude); experimental years; and some other parameters. In the cases that seasonal water supply were not presented directly, it was back-calculated from total seasonal precipitation, irrigation amounts and soil water supply. Similarly, in cases in which WP was not explicitly reported, the ratio of yield to water supply was considered to be the indirect WP. In addition to recording these data from each study, we also collected some parameters having high frequency in the cited literature and relating factors that affect WP for further data analysis: seasonal irrigation amounts; seasonal precipitation, soil properties (SOM, soil texture, soil pH) (Supplementary Database).

In further data compiling prior to analysis, we categorized the following factors: seasonal precipitation, irrigation amounts under different seasonal precipitation, soil organic matter, soil bulk density, soil pH, and deficit irrigation degree. Seasonal precipitation was categorized as low (<200 mm), medium (200–400 mm), and high (>400 mm) groups. Irrigation amounts under three different precipitation levels were taken into consideration due to the irrigation amount depending on the precipitation. The deficit irrigation data had to have the same managements and in pairs for every study; deficit irrigation rates were grouped into six brackets, as follows: <30%; 30–50%; 50–60%; 60–70%; 70–80%; and 80–100%.

### Definitions

Seasonal water supply (ET) was defined as in-season precipitation plus irrigation and changes in soil water content between sowing and harvest at a soil depth of at least 1 m.

Water productivity (WP) was defined as:

$$WP=\frac{Y}{ET}$$

where Y is maize yield (kg ha^−1^) and ET is seasonal water supply (mm). The unit of WP is kg ha^−1^ mm^−1^, which can be unified with the unit kg m^−3^ as follows:

$$W{P}_{kgh{a}^{-1}m{m}^{-1}}=W{P}_{kg{m}^{-3}}\times 10$$

### WP and yield gap analysis

Quantile regression^62^ was used to determine the WP boundary functions for the relationship between maize yield and seasonal water supply using IBM SPSS Statistics 19.0. First, all data were grouped into five regions depending on experiment location: Asia, North America, South America, Europe and Africa. Next, we ranked yield data based on the seasonal water supply, using 20 mm intervals. We selected the 95^th^ percentile of yield data points as the upper boundary. Detailed methods are provided in Lin and Liu^45^. The slope line indicates the highest yield at a given seasonal water supply level; in other words, the potential attainable WP (WP_P_). Because yield does not always increase with seasonal water supply, the boundary function follows a linear plateau model^63^. We selected the data points for maximum yield as the yield plateau. The fitted boundary function was:

1 $${\rm{YP}}={{\rm{WP}}}_{{\rm{P}}}\times ({\rm{seasonal}}\,{\rm{water}}\,{\rm{supply}}-{\rm{soil}}\,{\rm{evaporation}}),\,{\rm{if}}\,{\rm{seasonal}}\,{\rm{water}}\,{\rm{supply}}\ge n({\rm{mm}})$$

2 $${{\rm{Y}}}_{{\rm{P}}}={\rm{plateau}}\,{\rm{value}},\,{\rm{if}}\,{\rm{seasonal}}\,{\rm{water}}\,{\rm{supply}}\ge n\,({\rm{mm}})$$

where WP_P_ represents the highest attained water productivity (kg ha^−1^ mm^−1^) for that level of seasonal water supply, Yp represents potential yield (kg ha^−1^), and *n* represents the water supply level at the beginning of the Y_P_ plateau zone.When seasonal water supply <*n* (mm), Y_P_ was regarded as water-limited potential yield. Conversely, when seasonal water supply ≥*n* (mm), Y_P_ was regarded as non-water-limited potential yield. Yield gaps (Y_g_) were defined as the difference between potential yield and current actual yield based on field experiments. The Y_g_ values under water-limited and non-water-limited conditions were calculated for each field by formulas (1 and 2), based on field-specific seasonal water supply and the actual yield reported for each field^30^. The WP gap was defined as the difference between the maximum attainable WP and the currently achieved WP at the field scale.

### Data analysis

We performed a Kolmogorov-Smirnov test and determined that the distribution of yield and WP was not normal (*P* < 0.0001) accordingly, we used nonparametric procedures for further analysis. In this study, reports including deficit irrigation and tradition irrigation treatments from the 473 previous studies (1,028 paired measurements) were separately analyzed using the meta-analysis method to evaluate the general response of yield and WP to different deficit irrigation levels. Following previous work^64^, we calculated the natural log of the response ratio to evaluate the effect sizes in our study. We used an unweighted resampling method based on a fixed-effects model, and corrected for bias, with 95% confidence intervals (CIs) and a bootstrapping procedure using MetaWin version 2.0^65^. This was necessary because almost none of the studies we reviewed reported measures of variance. For ease of interpretation, we expressed the effect size as the percentage change, which has been estimated to be (R − 1) × 100%^66^. The results were considered significantly different from zero if the CI did not overlap with zero. Positive percentage changes indicated increases in the response variable under deficit irrigation, while negative values indicated decreases in the response variable.

In addition, the available key factors from 473 studies including seasonal precipitation (n = 3,274), both seasonal irrigation amounts and precipitation amounts (n = 2,924), soil organic matter (n = 1,780), soil bulk density (n = 2.093), and soil pH (n = 2,172) were used to analyze how the yield and WP responded to those influencing factors. We performed Pearson’s correlation analysis using IBM SPSS Statistics 19.0 to identify these factors influencing maize yield and WP. We created figure 1 with ArcGIS 10.2, and other figures were created using SIGMAPLOT version 12.5 software.

### Scenario analysis

Global maize production in 2005 was used as the reference scenario (S0). We analyzed three main scenarios for the target year 2050: no improvements in WP (S1 scenario), which was defined as already-realized WP; 50% of potential WP (S2 scenario), which was defined as the most easily achieved WP value, because of there were many study regions (204 site-years) in which even higher WP has already been accomplished; and achievement of 85% of potential WP (S3 scenario), which was defined as our WP target in future, because further gains require the elimination of small imperfections in the integrated management when average farm WP approaches the WP potential threshold. The global crop demand in 2050 was calculated by multiplying per capita demand by the total population forecast for 2050 by the United Nations (UN) Population Division’s median variant projection^6^. Maize production in 2050 is expected to double from 2005 levels^6^.

## Electronic supplementary material

Supplementary information

Supplementary databases
