# Supplementary material for: Closing water productivity gaps to achieve food and water security for a global maize supply
Source: Sci Rep. 2018 Oct 3;8:14762. doi: 10.1038/s41598-018-32964-4 (PMC6170377; doi:10.1038/s41598-018-32964-4)
Supplement: Supplementary file 1 — Supplementary information [file 41598_2018_32964_MOESM1_ESM.pdf]

Supplementary information

Closing water productivity gaps to achieve food and water security for a global maize supply

Huifang Zheng[1], Qianqian Bian[2], Yulong Yin[2], Hao Ying[2], Qinghua Yang[1*], Zhenling Cui[2*]

[1]College of Agronomy, Henan Agricultural University, Zhengzhou 45006, China,

[2] College of Resources & Environmental Sciences, Key Laboratory of Plant-Soil Interactions, Ministry of Education, China Agricultural University, Beijing 100193, China.

*Corresponding author: Qinghua Yang and Zhenling Cui. Email: yangqh2000@163.com; zhenlingcui@163.com

Supplementary table 1: The mean, maximum and minimum water productivity (WP) value of irrigation maize in 31 countries from global scale.

Countries	WP (kg ha^{-1} mm^{-1})			Yield (kg ha^{-1})		
	Mean	Maximum	Minimum	Mean	Maximum	Minimum
America	15.9	40.2	3.5	11155	17250	1100
Argentina	22.9	50.4	9.4	11202	16966	4490
Australia	19.0	28.6	9.0	9431	14200	5880
Bangladesh	6.5	8.9	4.9	7969	8840	6340
Brazil	20.6	28.8	9.3	12206	17500	3530
Britain	16.6	22.6	10.4	5325	6700	3700
Bulgaria	33.0	76.1	7.7	8450	14340	3838
China	21.9	53.7	3.6	9465	17998	908
Egypt	11.3	25.5	3.3	6266	12857	1291
France	19.1	21.7	14.8	11347	13800	7950
Germany	38.8	48.8	31.5	9884	11250	6653
Ghana	9.0	10.4	7.6	4215	4880	3550
India	15.0	30.1	5.1	6219	11320	1480
Iran	11.2	28.5	6.3	7285	11945	2923
Italy	25.0	39.5	13.4	12045	19778	5000
Lebanon	17.4	18.8	15.4	12125	14500	10400
Libyan	11.6	18.1	6.0	6023	10100	2864
Malawi	10.4	48.8	2.6	4679	6420	2370
Mozambique	6.1	11.9	2.4	2428	4800	940
Niger	5.4	7.3	2.6	2393	4506	609
Nigeria	5.0	21.6	3.1	2886	8330	1560
Pakistan	12.7	26.1	3.2	6014	10690	2160
Philippines	13.6	18.1	9.0	9300	10400	8200
Romania	14.9	18.9	9.1	9732	12473	6127
Serbia	31.4	39.0	23.7	14010	16330	12460
South Africa	21.4	28.8	10.9	8857	12429	3740
Spain	20.0	37.3	2.0	10738	18866	1030
Tanzania	6.1	8.5	4.1	2866	4350	1620
Turkey	16.8	28.3	4.4	9969	18060	1440
Uzbekistan	5.0	7.7	3.4	1950	2870	1390
Zambia	9.0	13.2	3.7	3673	6000	1500

Supplementary table 2: Data from FAO showing the ratio of irrigation area harvested (every country/ 31 counties) for the top five maize production countries, together with the percentage of data points in the meta-analysis that come from those countries.

Country	% irrigation area	% data points
China	52	47.9
United States of America	19	29.2
India	5.6	3.3
Pakistan	3.8	2.3
Egypt	3.7	3.2

Detailed sources on data resource used for the meta-analysis

1. He, F.B. & Sun A.L. Influence on water consumption law and maize yield by alternative furrow irrigation. *Heilongjiang Science and Technology of Water Conservancy* **40**, 5-8 (2012) (in Chinese with English abstract).
2. Yuan, W.K. Study on effects of water-saving and increase production under the covering film with spray irrigation in western sandy area of Hei Long Jiang. *Dissertation for the Master Degree, Northeast Agricultural University* (2010) (in Chinese with English abstract).
3. Sun, A.L. Study on water technique of maize for alternative furrow irrigation in semi-arid region of western Hei Long Jiang. Province. *Dissertation for the Master Degree, Northeast Agricultural University* (2011) (in Chinese with English abstract).
4. Zhao, Y.Y. The experimental study on furrow irrigation pattern of corn in semi-arid region of western Hei Long Jiang. Province. *Dissertation for the Master Degree, Northeast Agricultural University* (2011) (in Chinese with English abstract).
5. Deng, C.H., Wang M.X. & Wang N. Effects of different water and fertilizer group on water use efficiency and economic benefit in maize. *Hei Long Jiang Agricultural Sciences* **3**, 63-65(2008) (in Chinese with English abstract).
6. Li, Y.X., Wei Y.H &Wei Y.X. Effect of Conservation Tillage on soil moisture and corn yield. *China Rural Water and Hydropower*, 25-28 (2010) (in Chinese with English abstract).
7. Li, N.N. Coupling effects between water and nitrogen of corn on drip irrigation under plastic film in semiarid region of Hei Long Jiang province. *Dissertation for the Master Degree, Northeast Agricultural University* (2010) (in Chinese with English abstract).
8. Liu, Y.L. The studies on under mulch drip effects between water saving yield and temperature improving of maize in west semiarid region of Hei Long Jiang province. *Dissertation for the Master Degree, Northeast Agricultural University* (2010) (in Chinese with English abstract).
9. Lv, G.L. Study on the effects of water saving and increasing yield under the different film mulching methods and drip irrigation. *Dissertation for the Master Degree, Northeast Agricultural University* (2011) (in Chinese with English abstract).
10. Li, Y.K. Study on the effects of water and fertilizer on maize in the west semiarid region of Hei Long Jiang province. *Dissertation for the Master Degree, Northeast Agricultural University* (2009) (in Chinese with English abstract).
11. Wang, Y.X. Effect of different irrigation method on yields of film-mulching maize in semi-arid area. *Hei Long Jiang Agricultural Sciences* **3**, 22-25 (2014) (in Chinese with English abstract).
12. Gao, Y.S., Liu, H.T., Bian, X.Z., Dou, J.G., Sun,Y. & Ren, J. the suitable amount of potassium fertilizer of maize under chernozem conditions in Western Jilin Province. *Journal of Jinlin Agricultural Sciences* **31**, 39-41 (2006) (in Chinese with English abstract).
13. Wang, J.D., Zhang, Y.Q., Gong, S.H., Xu, D. & Zhao, Y.F. Study on the application

effects of shallow subsurface drip irrigation mode under mulch. *Journal of Irrigation and Drainage* **34**, 1-5 (2005) (in Chinese with English abstract).
14. Wang, L.M., Wei, Y.X., Wang, C. &Wang, B. Water-saving and anti-drought combined technological measures' influences on maize yield formation factors and water utilization efficiency. *Journal of Irrigation and Drainage* **24**, 23-26 (2005) (in Chinese with English abstract).
15. Meng, K., Zhang, X.Y., Sui,Y.Y. & Zhao J. The crop yields and water use efficiency under different water fertilizer condition in the field of black soil. *Chinese Journal of Eco-Agriculture* **13**, (2005) (in Chinese with English abstract).
16. Meng, K. & Zhang, X.Y. Study on crop water use efficiency in black soil regions of Northeastern China. *Eco-Agricultur research* **13**, 119-121 (2005) (in Chinese with English abstract).
17. Cao, Q.J., Cui, J.H., Wang, H.Y., Wen, H.J., Gao, Y.N., Luo, L.H. & Han, H.F. Effect of water treatments after jointing stage on plant characters and water use efficiency of maize. *Journal of Maize Sciences* **19**, 105-109 (2011) (in Chinese with English abstract).
18. Zhang, Y. Effects of the water-retaining agent on growing development and evapotranspiration of spring corn and soybean. *Dissertation for the Master Degree, Northeast Agricultural University* (2013) (in Chinese with English abstract).
19. Liu, Y., Li, Y.F., Li, J.S. &Yan, H.J. Effects of mulched drip irrigation on water and heat conditions in field and maize yield in sub-humid region of Northeast China. *Journal of agricultural machinery* **46**, 93-104 (2015) (in Chinese with English abstract).
20. Cao, Y.J. *et al*. Effects of different films on soil water, temperature and corn growth characteristics under drip-irrigation conditions in semi-arid region. *Journal of Maize Sciences* **21**, 107-113 (2013) (in Chinese with English abstract).
21. Guo, W. The experimental study of corn growing using drip irrigation under plastic membranes in Northwestern area Heilongjiang Province. *Dissertation for the Master Degree, Northeast Agricultural University* (2010) (in Chinese with English abstract).
22. Ji J.H., Li, Y.Y., Liu, S.Q & Tong, Y.X. Effect of Drip Irrigation under plastic film mulch on photosynthetic, dry matter accumulation and water use efficiency. *Journal of Maize Sciences* **23**, 128-133 (2015) (in Chinese with English abstract).
23. Li, E, X. The studies on under mulch drip irrigation system of maize in western region of Hei Long Jiang Province. *Dissertation for the Master Degree, Northeast Agricultural University* (2015) (in Chinese with English abstract).
24. Xu, J. et al. The effects of plastic film mulching on maize growth and water use in dry and rainy years in northeast china. *Plos One* **10**, e125781 (2015).
25. Xu, J. The Effects of plastic film mulch and drip irrigation on spring maize yield and water-nitrogen use efficiency in Northeast China. *Dissertation for the Ph.D. Degree, China Agricultural University* (2015) (in Chinese with English abstract).
26. Kang, Z.J. *et al*. The relationship between corn yield and water aided by Cropwat model. *Journal of Maize Sciences* **18**, 114-117 (2010) (in Chinese with English abstract).

27. Liu, Y., Sun, Z.X., Bai, Y., Zheng, J.M., Hou, Z.Y., Zhang, Y & Weng, F. Effect of maize and soybean inter-planting on cops growth and yield in Western Liaoning province. *Soybean Science* **30**,224-228 (2011) (in Chinese with English abstract).
28. Yin, G.H., Shen, Y.J., Zhang, F.S., Tong, N. & Liu Z.X. Patterns of techniques integration for water-saving and high yield on spring maize in semi-arid area of West Liao Ning. *Chinese Agricultural Science Bulletin* **26**, 177-180 (2010) (in Chinese with English abstract).
29. Yin, G.H., Shen, Y.J., Kang, Z.J., Zhang, F.S. & Liu Z.X. Screening on varieties of spring maize in semiarid region of West Liaoning. *Chinese Agricultural Science Bulletin* **27**, 195-198 (2011) (in Chinese with English abstract).
30. Wang, Y.M., Yin, G.H., Wang, X. & Wu, G.W. A preliminary study on limited supplementary irrigation system of corn in the semi-arid area of Western Liao Ning. *Modern Agricultural Sciences* **15**, 108-110 (2008) (in Chinese with English abstract).
31. Zhang, D. & Gong S.H. Research on the influence of mulch-drip irrigation and no-mulch drip irrigation of soil moisture and maize yield-a case study of the Western region of Liao Ning province. *China Rural Water and Hydropower,* 9-13 (2016) (in Chinese with English abstract).
32. Dou, C.Y. & Meng, W.Z. Effect of planting density on growth and yield of maize in double line at one width ridge under mulch-drip irrigation. *Journal of Irrigation and Drainage* **33**, 97-100 (2014) (in Chinese with English abstract).
33. Dou, C.Y. & Meng, W.Z. Application of controlled irrigation on maize planted in double row at one wide ridge with mulch-drip irrigation. *Journal of Jilin Agricultural Sciences* **39**, 16-19 (2014) (in Chinese with English abstract).
34. Dou, C.Y. & Meng, W.Z. Research o application of mulch-drip irrigation under different landform condition inn semi-arid area of West Liao Ning. *Water-Saving Irrigation*, 19-21 (2014) (in Chinese with English abstract).
35. Li, K.Y., Zheng, J.M., Sun, Z.X., Yang, N., Feng, L.S. & Zhang, Z. Screeningand Evaluation of High Water Use Efficiency Maize varieties with drip irrigation under mulching plastic film in western Liao Ning province. *Hubei Agricultural Sciences* **54**, 1560-1563 (2015) (in Chinese with English abstract).
36. Li, Z., Yu, X.Q., Chen, Y. & Liu A.M. Study on irrigation schedule of maize in western Liao Ning province. *Modern Agricultural Sciences* **584**, 21-22 (2012) (in Chinese with English abstract).
37. Chen, S.T. & Zheng, X. L. The effect of different tillage methods on soil moisture content and water use efficiency. *Anhui Agriculture Sciences Bulletin* **14**, 60-61 (2008) (in Chinese with English abstract).
38. Yu, M., Zhen, W.C., Wang, Z.Y. & Wen, H.D. Response of two corn varieties to water and density in piedmont. *Water-Saving Irrigation*, 11-14 (2010) (in Chinese with English abstract).
39. Li, C.H., Zhao, X., Liu, T.X. & Kang, B. M. Effects of different treatments of winter wheat residues on eco-physiological responses of mechanized sowing summer maize (*Zea mays* L.). *Transactions of the Chinese Society of Agricultural Engineering* **24**, 162-166 (2008) (in Chinese with English abstract).

40. Wang, H.Y., Hu, K.L., Li, B.G. & Jin, L. Analysis of water and nitrogen use efficiencies and their environmental impact under different water and nitrogen management practices. *Scientia Agricultural Sinica* **44**, 2701-2710 (2011) (in Chinese with English abstract).
41. Zhang, H.M. Water and Nitrogen Dynamics in soil and crops growth as affected by sprinkle irrigation and fertilization management. *Dissertation for the Master Degree, Agricultural University of Hebei* (2009) (in Chinese with English abstract).
42. He, J., Wang, Q., Li, H., Liu, L. & Gao, H. Effect of alternative tillage and residue cover on yield and water use efficiency in annual double cropping system in north china plain. *Soil Till Res* **104**, 198-205 (2009).
43. Jin, L., Hu, K.L., Li, B.G. & Gong, Y.S. Coupled simulation on crop growth and soil water-heat-nitrogen transport-Model validation and application. *ShuiLi XuBao* **38**, 972-980 (2007) (in Chinese with English abstract).
44. Zhou, X.L. Optimization of water resources utilization for cropping system in Beijing. *Dissertation for the Ph.D. Degree, China Agricultural University* (2005) (in Chinese with English abstract).
45. Li, P. & Li, J.S. Effects of drip irrigation placement depth on the accumulation of assimilates and water and nitrogen use efficiency of Spring Maize. *Conference paper* (2011) (in Chinese with English abstract).
46. He, J., Wang, Q.J., Li, H.W., Lv, C.Y., Qiao, X.D. & Lu, Z.Y. Effects of no-till opening seedbed on crop growth in annual double cropping areas in Northern China. *Journal of agricultural machinery* **44**, 50-56 (2013) (in Chinese with English abstract).
47. He, J. et al. Permanent raised beds improved crop performance and water use on the north china plain. *J Soil Water Conserv* **70**, 54-62 (2015).
48. Tan, J.L. Field experimental study on summer corn water consumption and growths in Beijing. *Dissertation for the Ph.D. Degree, Yangzhou Agricultural University* (2013) (in Chinese with English abstract).
49. Lv, G.H., Kang, Y,H. & Li, L. Effect of sprinkler irrigation on water, grain yield and water use efficiency in the winter wheat and summer maize rotating system *Conference paper* (2009) (in Chinese with English abstract).
50. Zha, G.F., Huang, G.H., Feng, S.Y. & Qi, Z.M. Water and nitrogen use efficiency for summer corn under condition of irrigation with sewage effluent. *Transactions of the Chinese Society of Agricultural Engineering* **19**, 63-67 (2003) (in Chinese with English abstract).
51. Liu, Y.N. & Zeng, Q. Analyses for drought-resistance of current spread hybrids of maize in China. *Acta Agriculturae Boreali Sinica* **10**, 45-50 (1999) (in Chinese with English abstract).
52. Gao, Y.B., Tao, H.B., Zhu, J.C., Huang, S.B., Xu, C.L., Sheng, Y.H. & Wang P. Effects of wheat stubble height on growth and water use efficiency of mechanized sowing summer maize. *Scientia Agricultural Sinica* **48**, 3803-3810(2015) (in Chinese with English abstract).
53. Guo, B.Q., Tao, H.B., Wang P., Knoerzer, H.K. & Claupein, W. Water utilization of different cropping production systems in North China Plain. *Journal of China*

Agricultural University **18**, 53-60 (2013) (in Chinese with English abstract).
54. Liu, M., Tao, H.B., Wang P., Lv, L.H. & Zhang, Y.J. Analysis of different cropping systems using optimizing water-nitrogen management on yield , water and nitrogen utilization and economic benefits. *Journal of China Agricultural University* **13**, 12-18 (2008) (in Chinese with English abstract).
55. Kong, F.L., Zhang, H.L., Zhai, Y.L Yuan, J.C. & Chen. F. Effects of tillage methods on crop yield and water use characteristics in winter-wheat/summer-maize rotation system in the North China Plain. *Chinese Journal of Eco-Agriculture* **22**, 749-756 (2014) (in Chinese with English abstract).
56. Yi, Z.W., Wang, P., Chen, P.P., Tu, N.M. & Lan, L.W. Application of coated urea in summer maize in North China Plain. *Acta Ecological Sinica* **28**, 4919-4928 (2008) (in Chinese with English abstract).
57. Liu, M., Tao, H.B., Wang P. & Zhang, Y.J. Effects of sowing date on growth, yield formation and water utilization of spring maize. *Journal of Maize Sciences* **17**, 108-111 (2009) (in Chinese with English abstract).
58. Zhu, J.C., Tao, H.B., Gao, Y.B., Ming, B. & Wang P. Effects of sowing irrigation on plant establishment ,water consumption and yield of summer maize. *Journal of China Agricultural University* **18**, 34-38 (2013) (in Chinese with English abstract).
59. Lv, L., Wang, H., Jia, X. & Wang, Z. Analysis on water requirement and water-saving amount of wheat and corn in typical regions of the north china plain. *Frontiers of Agriculture in China* **5**, 556-562 (2011).
60. Ma, L., Liu, T.X., Han, D.G., Fu, J., Zhao, Z.J. & Li, C.H. Effects of ridge culture on grain yield and water use efficiency of winter wheat and summer maize. *Journal of Nuclear Agricultural Science* **24**, 1062-1067 (2010) (in Chinese with English abstract).
61. Ma, L. Research on eco-physiological effects of integrative ridge cultivation practice on winter wheat and summer maize. *Dissertation for the Master Degree, Henan Agricultural University* (2007) (in Chinese with English abstract).
62. Song, L.L. Study on field water consumption law and irrigation effect of winter wheat and summer corn continuous cropping in the irrigation area of north of Henan province. *Dissertation for the Master Degree, Henan Agricultural University* (2007) (in Chinese with English abstract).
63. Feng, Y., Yin, B,Z., Ma, Y.H., Guo, L.G. & Zhen, W.C. Influence of integration of subsoiling and sowing on root growth of maize and water utilization of farmland. *Journal of Agricultural University of Hebei* **37**, 1-6 (2014) (in Chinese with English abstract).
64. Wang, L., Du, X., Cui, Y.L., Dang, H.K., Li, K.J., He, Z.Y. & Yu, X.Y. Effects of removing redundant organs on yield formation of high-yielding summer maize. *Acta Agriculturae Boreali Sinica* **30**, 132-138 (2015) (in Chinese with English abstract).
65. Dang, H.K., Li, W., Cao, C.Y., Zheng, C.L., Ma, J. Y. & Li K.J. Effects of Late Milk Irrigation on Water Use Efficiency and Dry Matter Distribution of Maize. *Journal of agricultural machinery* **45**, 131-138 (2014) (in Chinese with English abstract).

66. Tuo, Y.F. Study on water & heat transfer model and high efficient water using techniques of summer corn straw mulching in Hebei province. *Dissertation for the Master Degree, Henan Agricultural University* (2006) (in Chinese with English abstract).
67. Wang, L. The approaches for exploring summer maize yield and study on appropriate cropping system in Hebei Plain. *Dissertation for the Master Degree, Henan Agricultural University* (2015) (in Chinese with English abstract).
68. Feng, Y. Effect of changes in soil water and heat on summer maize root under subsoiling seed condition. *Dissertation for the Master Degree, Henan Agricultural University* (2014) (in Chinese with English abstract).
69. Dai, X.Q., Sui, P. & Xie, G.H. Water use and nitrate nitrogen changes in intensive farmlands following introduction of poplar (populus x euramericana) in a semi-arid region. *Arid Land Res Manag* **20**, 281-294 (2006).
70. Dong, X.W., Liu, P.L., Liu, S.T., Liu, S.L. & Liu, P.Q. Study on water consumption characteristics and irrigation indexes of summer maize. *Journal of Maize Sciences* **5**, 53-57(1996) (in Chinese with English abstract).
71. Li, H.R. Effects of fertigation on winter wheat-summer maize growth and water and nitrogen use efficiency. *Dissertation for the Master Degree, China Agricultural University* (2013) (in Chinese with English abstract).
72. Sun, W.Y. Effect of types and rates of nitrogen fertilizers on yield different wheat and maize varieties and corresponding water and nitrogen use efficiencies. *Dissertation for the Ph.D. Degree, Chinese Academy of Agricultural Sciences* (2013) (in Chinese with English abstract).
73. Wang, R.Y., Wang, S.J., Wang, Y.F., Du, J.Z. & Zhao, C.X. Screening of varieties in wheat-maize biological water-saving annual culture. *Chinese Agricultural Science Bulletin* **26**, 157-162 (2010) (in Chinese with English abstract).
74. Wang, H.L. Design and Vali-dating sustainable maize (*Zea mays* L.) system under climate change in the North China plain. *Dissertation for the Master Degree, China Agricultural University* (2014) (in Chinese with English abstract).
75. Meng, Q.F. et al. Alternative cropping systems for sustainable water and nitrogen use in the north china plain. *Agr Ecosyst Environ* **146**, 93-102 (2012).
76. Zheng, L. Spatio-temporal patterns of soil organic matter and optimal management for water and nitrogen in typical high-yielding grain crop field of Northern China. *Dissertation for the Ph.D. Degree, Chinese Academy of Agricultural Sciences* (2014) (in Chinese with English abstract).
77. Chang, X., Wei, K.M., Wang, H.Z., Yang, S.J., Shao, Y., Qi, S. & Wang, T.C. Effects of different tillage on soil water content of summer maize. *Journal of Irrigation and Drainage* **31**, 75-78 (2012) (in Chinese with English abstract).
78. Qiang, X.M., Sun, J.S., Fan, X.Y. & Zhang, X.P. Effects of mulching on soil water dynamics and yield of summer. *Journal of Irrigation and Drainage* **31**, 42-45 (2014) (in Chinese with English abstract).
79. Gao, Y. Transport and use of PAR and water in maize/soybean strip intercropping system. *Dissertation for the Ph.D. Degree, Chinese Academy of Agricultural Sciences* (2014) (in Chinese with English abstract).

80. Jiao, W.H. The simulation research of a long-term soil water and salt transport and crop growth under saline water irrigation in Hengshui region. *Dissertation for the Master Degree, China University of Geosciences* (2015) (in Chinese with English abstract).
81. Zhang, X.Y., Pei, D. & Hu, C.S. Index System for irrigation scheduling of winter wheat and maize in the piedmont of mountain Taihang. *Transactions of the Chinese Society of Agricultural Engineering* **18**, 36-41 (2002) (in Chinese with English abstract).
82. Pei, D., Chen, S.Y. & Zhang, X.Y. Optimized irrigation scheduling for maize in the piedmont of mountain Taihang of the North China Plain. *Chinese Journal of Eco-Agriculture* **12,** 144-147 (2004) (in Chinese with English abstract).
83. Sun, H.Y., Zhang, X.Y., Chen, S.Y., Pei, D. & Liu, C.M. Effects of harvest and sowing time on the performance of the rotation of winter wheat-summer maize in the north china plain. *Ind Crop Prod* **25**, 239-247 (2007).
84. Zhang, Y.Q. et al. Effect of soil water deficit on evapotranspiration, crop yield, and water use efficiency in the north china plain. *Agr Water Manage* **64**, 107-122 (2004).
85. Zhang, X., Chen, S., Liu, M., Pei, D. & Sun, H. Improved water use efficiency associated with cultivars and agronomic management in the north china plain. **v. 97** (2005).
86. Zhang, X.Y., Pei, D., Chen, S.Y., Sun, H.Y. & Yang, Y.H. Performance of double-cropped winter wheat-summer maize under minimum irrigation in the north china plain. *Agron J* **98**, 1620-1626 (2006).
87. Zhang, X.Y., Chen, S.Y., Sun, H.Y., Shao, L.W. & Wang, Y.Z. Changes in evapotranspiration over irrigated winter wheat and maize in north china plain over three decades. *Agr Water Manage* **98**, 1097-1104 (2011).
88. Sun, H.Y., Zhang, X.Y., Chen, S.Y. & Shao, L.W. Performance of a double cropping system under a continuous minimum irrigation strategy. *Agron J* **106**, 281-289 (2014).
89. Zhang, X., Pei, D. & Hu, C. Conserving groundwater for irrigation in the north china plain. *Irrigation Sci* **21**, 159-166 (2003).
90. Hongyong, S. et al. Effect of precipitation change on water balance and wue of the winter wheat-summer maize rotation in the north china plain. *Agr Water Manage* **97**, 1139-1145 (2010).
91. Wang, M. Research about agronomic characters and water use efficiency of 10 summer maize varieties. *Dissertation for the Master Degree, Northwest University of agriculture and Forestry* (2012) (in Chinese with English abstract).
92. Li R.X. Effects of different irrigation strategies on crop yield and WUE in winter wheat-summer maize system. *Dissertation for the Master Degree, Agriculture University of Hebei* (2010) (in Chinese with English abstract).
93. Hou, D.S., Li, Y.H., Zhang, J., Tian, H.W., Li, J.R., Feng, L.H., He, J.L., Li, J.B., Ding, Y.F., Sun, M.Q. & Lin, X.C. Effect of straw returned on wheat-corn yield and fluctuation of water use efficiency. *Chinese Journal of Eco-Agriculture* **19,** 41-44 (2015) (in Chinese with English abstract).

94. Li, G.Q., Zhou, J., Xu, P., Huang, Z.J. & Zhang, Z.B. Correlation and path coefficient analysis in maize ear traits of drought and water saving. *Acta Agriculturae Boreali Sinica* **24**, 131-136 (2015) (in Chinese with English abstract).
95. Wang, M., Xu, P., Liu, X.J., Zhang, Z.B. & Yang, Y.F. Path analysis of summer maize agronomic traits and yield in the Huang-Huai-Hai Plain. *Chinese Journal of Eco-Agriculture* **19,** 1229-1236 (2011) (in Chinese with English abstract).
96. Chen, S.Y., Zhang, X.Y., Hu, C.S. & Liu M. Y. Research on the spatial variability of soil water and salt and rational sampling number. *Agricultural Research in the Arid Areas* **20**, 55-57 (2002) (in Chinese with English abstract).
97. Chen, S.Y., Zhang, X.Y., Pei, D. & Sun H. Y. Soil evaporation and soil temperature in maize field mulched. *Journal of Irrigation and Drainage* **23**, 32-36 (2004) (in Chinese with English abstract).
98. Shao, L.W., Zhang, X.Y., Chen, S.Y., Sun H. Y.& Pei, D. Yield and water use efficiency affected by rainfall ,irrigation and maize varieties. *Journal of Irrigation and Drainage* **28**, 48-51 (2009) (in Chinese with English abstract).
99. Lin, M. Field experimental study on the growth of summer corn under different tillage. *Dissertation for the Master Degree, Yangzhou Agricultural University* (2014) (in Chinese with English abstract).
100. Shao, G.Q., Li, Z.J., Ning, T.Y., Zheng, Y.H., Tian, S.Z. & Wang, Y. Effects of irrigation and urea types on water use efficiency of maize. *Transactions of the Chinese Society of Agricultural Engineering* **26**, 58-63 (2010) (in Chinese with English abstract).
101. Li, Z.J. Evaluating nitrogen loss and water and nitrogen use efficiencies in a double cropping system under different integrated managements in the North China Plain. *Dissertation for the Ph.D. Degree, China Agricultural University* (2015) (in Chinese with English abstract).
102. Shao, G.Q. Study on the coupling effects of controlled release urea and water on nitrogen and water utilization yield and quality of maize. *Dissertation for the Master Degree, Shangdong Agricultural University* (2008) (in Chinese with English abstract).
103. Wang, X.D., Wang, C.T., Hou, H.H., Yang, Q.S & Wang, S. The soil moisture and water use efficiency of summer maize under pipe-evenly-moving precision irrigation. *Water-Saving Irrigation*, 22-26 (2014) (in Chinese with English abstract).
104. Wu, J.C., Yang, Y.H., Zheng, H.L., Li, X.J., He, F. & Han, W.F. effect of stage irrigation and fertilizer coupling on crop yield and water utilization in sandy land. *Journal of Irrigation and Drainage* **33**, 35-39 (2014) (in Chinese with English abstract).
105. Zhou, J.H. & Jia, S.T. Effects of different irrigation and fertilization methods on yield and water use efficiency of spring corn. *Chinese Agricultural Science Bulletin* **29**, 224-227 (2013) (in Chinese with English abstract).
106. Zhao, Y.L., Xue, Z.W., Guo, H.B., Mu, X.Y. & Li, C.H. Effects of tillage and straw returning on water consumption characteristics and water use efficiency in the winter wheat and summer maize rotation system. *Scientia Agricultural Sinica* **47**, 3359-3371 (2014) (in Chinese with English abstract).

107. Dai, K. optimization of water and nitrogen use efficiencies in winter wheat and summer maize cropping system in North China Plain. *Dissertation for the Ph.D. Degree, Chinese Academy of Agricultural Sciences* (2012) (in Chinese with English abstract).
108. Liu, X.F., Sun, J.S., Liu, Z.G., Wang, J.L., Zhang, J.Y. & Zhang, J.P. Effects of mulching on plant growth yield of summer maize under sprinkler irrigation. *Journal of Maize Sciences* **19**, 113-117(2011) (in Chinese with English abstract).
109. Du, T., Kang, S., Sun, J., Zhang, X. & Zhang, J. An improved water use efficiency of cereals under temporal and spatial deficit irrigation in north china. *Agr Water Manage* **97**, 66-74 (2010).
110. Gao, Y. et al. Crop coefficient and water-use efficiency of winter wheat/spring maize strip intercropping. *Field Crop Res* **111**, 65-73 (2009).
111. Zhou, X.G., Li, C.X. Qiang, X.M. & Guo, D.D. Effects of liquid film mulching on dry matter accumulation and water use efficiency of maize with sprinkler irrigation. *Transactions of the Chinese Society of Agricultural Engineering* **26**, 43-48 (2010) (in Chinese with English abstract).
112. Shao, L.W., Wang, Y.Z., Miao, W.F., Sun, H.Y., Chen, S.Y. & Zhang, X.Y. Effect of cultivar and plant density on summer maize grain yield and water use efficiency in North China Plain. *Acta Agriculturae Boreali Sinica* **26**, 182-188 (2011) (in Chinese with English abstract).
113. Su, P.F., Zhao, S.Y., Song, S.B. & Shi, Q. Effects of no-tillage on water use efficiency in inter-planted summer corn. *Journal of Irrigation and Drainage* **18**, 32-35 (1999) (in Chinese with English abstract).
114. Zhang, J.P., Liu, Z.G., Sun, J.S., Feng, L. & Liu, X.F. Effects of different moisture and mulching treatments on soil hydrothermal regimes and summer maize growth. *Journal of Irrigation and Drainage* **34**, 25-28 (1999) (in Chinese with English abstract).
115. Wang, C.X., Liu, Z.G., Wu, H.Q., Feng, S.Y., Huang, G.H. & Zhan, W.H. Suitable combination of water and fertilizers for inter-planted summer corn under drip irrigation. *Journal of Irrigation and Drainage* **18**, 17-208 (1999) (in Chinese with English abstract).
116. Yu, S.Z., Chen, Y.H., Li, Q.Q., Zhou, X.B., Fang, Q.X., Wang, J.S., Liu, E.M. & Luo, Y. Feasible study on water-saving effect of wheat-maize rotation pattern. *Acta Ecological Sinica* **26**, 2523-2531 (2006) (in Chinese with English abstract).
117. Zhang, H.L. Field Water Consumption and control of no-tillage with mulch soil-crop system for double cropping region in North China. *Dissertation for the Ph.D. Degree, China Agricultural University* (2001) (in Chinese with English abstract).
118. Fang, Q.X. et al. Much improved irrigation wheat-maize double use efficiency in an intensive cropping system in the north china plain. *J Integr Plant Biol* **49**, 1517-1526 (2007).
119. Fang, Q. et al. Irrigation strategies to improve the water use efficiency of wheat-maize double cropping systems in north china plain. *Agr Water Manage* **97**, 1165-1174 (2010).
120. Wang, T.C., Li, X.M., Sui, R.T. & Liu, D.J. A primary study on the technical effects

of subsoiling in row space at corn seedling growth stage. *Chinese Agricultural Science Bulletin* **19**, 40-43 (2003) (in Chinese with English abstract).
121. Meng, Z.Y. Studies on the compling effects of irrigation and nitrogen fertilization on summer corn in Zhengzhou region. *Dissertation for the Master Degree, Henan Agricultural University* (2008) (in Chinese with English abstract).
122. Wang, S.S., Liu, H., Liu, Z.T. & Meng, P.T. Huang, F.P. Water consumption characteristics and relationship yield and soil moisture of summer maize in wild ridge planting way. *Journal of Irrigation and Drainage* **34**, 62-66 (2015) (in Chinese with English abstract).
123. Wang, S.S., Liu, D.X., Wang, K.S & Meng, P.T. Fuzzy comprehensive evaluation on water consumption characteristics and yield of summer corn under different furrow irrigation patterns. Transactions of the Chinese Society of Agricultural Engineering **31**, 89-94 (2015) (in Chinese with English abstract).
124. Li, L., Zhu, C.C., Wang, T.C., Jiang, Y.M., Zhang, H.Q. & Huang, X.S. Effects of different planting patterns on soil moisture and summer maize yield. *Journal of Henan Agricultural University* **46**, 511-514 (2012) (in Chinese with English abstract).
125. Ma, H.Y., Zhang, Z.Y., Wang, X., Huang, Q. & Yu, X.L. Study on Growth， Yield and Water Use Efficiency of Summer maize under Film-furrow Irrigation. *Water-Saving Irrigation*, 52-54 (2012) (in Chinese with English abstract).
126. Li, Y.K., Xia, X., Chen, M.P., Wang, L.C. & Mei, X.R. Effects of fertilization increasing carbon on soil moisture and crop water use efficiency in North China Plain. *Journal of Irrigation and Drainage* **34**, 50-53 (2015) (in Chinese with English abstract).
127. Li, H.R., Mei, X.R., Hao, W.P., Xia, X., Liu, Q. & Li, Y.K. Effects of different irrigation and fertilizer regimes on soil moisture and grain yield of summer maize. *Journal of Irrigation and Drainage* **31**, 72-74 (2012) (in Chinese with English abstract).
128. Lin, L. Evaluation of nitrogen loss way, water and nitrogen use efficiencies in winter wheat and summer maize rotation system under different fertilizer n managements —a case study in Huantai county. *Dissertation for the Master Degree, Shangdong Agricultural University* (2011) (in Chinese with English abstract).
129. Ma, Y., Feng, S. & Song, X. A root zone model for estimating soil water balance and crop yield responses to deficit irrigation in the north china plain. Agr Water Manage 127, 13-24 (2013).
130. Xie, Y.H., Li, L., Hong, J.P., Wang, H.T. & Zhang, L. Effects of nitrogen application and irrigation on grain yield, water and nitrogen utilizations of summer maize. *Plant Nutrition and Fertilizer Science* **18**, 1354-1361 (2012) (in Chinese with English abstract).
131. Ma, L., Mao, Q.N., Li, Q., Cao, X.H. Chen, X.M. Research on summer maize water saving irrigation schedule under straw returning condition in Jinghuiqu irrigation district. *Water-Saving Irrigation*, 15-20 (2016) (in Chinese with English abstract).
132. Yin, B.Z., Zhen, W.C. Feng, Y. Effects of subsoiling-seeding on root physiological indices, water-saving and yield-increasing behaviors in summer maize (*Zea mays*

L.). *Acta Agronomica Sinica* **41**, 623-632 (2015) (in Chinese with English abstract).
133. Liang, Z.J., Qi, H.L., Wang, Y.X., Dong, P., Zhang, D.M., Yang, Y.B., Nan, X.Q., Zhao, H.Z. & Xi, T.Y. Effects of different drip irrigation quota on photosynthetic performance and water use efficiency of maize. *Chinese Agricultural Science Bulletin* **30**, 74-78 (2014) (in Chinese with English abstract).
134. Zhan, Q.H. & Chen, J. Effect of coordination of water and fertilizer on maize yield and theirs utilization efficiency. *Soils and Fertilizers*, 14-18 (2005) (in Chinese with English abstract).
135. Zhan, Q.H. Study on genetic features of vertisol arable land and its agricultural utilization technology. *Dissertation for the Master Degree, Nanjing Agricultural University* (2011) (in Chinese with English abstract).
136. Yang, Y.H., Wu, J.C., Wang, H.Q., Guo, Q., He, F. & Pan, X.Y. Effect of different measures of tillage and soil moisture conservation on anniversary water use efficiencies of wheat and corn. *Journal of Irrigation and Drainage* **33**, 63-66 (2014) (in Chinese with English abstract).
137. Yang, Y.H., Wu, J.C., Wang, H.Q., Guo, Q., He, F., Han, W.F. & Pan, X.Y. Effects of different water and fertilizer conditions on growth and water use efficiency of maize. *Journal of Henan Agricultural University* **44**, 50-54 (2015) (in Chinese with English abstract).
138. Yang, Y.H., Wu, J.C., Wang, H.Q., Zhao, L.J. & Guo, Q. Effects of nitrogen ferterlizer on growth and water use efficiency of maize in sandy-loam alluvial soil. *Journal of Henan Agricultural University* **42**, 52-58 (2013) (in Chinese with English abstract).
139. Hammad, H.M., Ahmad, A., Abbas, F. & Farhad, W. Optimizing water and nitrogen use for maize production under semiarid conditions. *Turk J Agric for* **36**, 519-532 (2012).
140. Qin, S.J. Study on yield and water effect and physiological properties of wheat and corn under supplementary irrigation with catchment rainfall in semi-Arid area of central Gansu. *Dissertation for the Master Degree, Gansu Agricultural University* (2000) (in Chinese with English abstract).
141. Zhang, X.C., Xia, F.Q. & Yang, F.K. Study on the effect of rainwater harvesting under the dryland film corn. *Gansu Agricultural Science and Technology*, 21-23 (2003) (in Chinese with English abstract).
142. Yin, G.H., Liu, Z.X., Lin, H.M. & Zhang. Z.S. Optimum period and amount of supplementary irrigation with catchment of rainfall on upland field with different cropping patterns. *Agricultural Research in the Arid Areas* **18**, 85-90 (2000) (in Chinese with English abstract).
143. Guo, Z.F., Lan, X.L. & Chen, F. Study on different cultivation ways of dry land soil conservation in mountain area of Southern Ningxia. *Ningxia Journal of Agricultural and Forest Science and Technology* **54**, 93-98 (2013) (in Chinese with English abstract).
144. Zhang, H.L., Liu, X.J., Wang, L. & Lv, Y. Study on technology of efficient utilization of rainfall resources in supplementary irrigated area of pumping from Yellow river in Ningxia. *Journal of Water Resources & Water Engineering* **24**, 119-

122 (2013) (in Chinese with English abstract).
145.Bu, Y.S. The farmland ecological effects and the crop yield-increasing mechanisms of different mulching materials. *Dissertation for the Ph.D. Degree, Shanxi Agricultural University* (2004) (in Chinese with English abstract).
146.Gui, L.G., Wang, P. & Wang, T.N. Research in limited irrigation technology of maize in the Changcheng irrigation regions. *Ningxia Journal of Agricultural and Forest Science and Technology* **52**, 62-64 (2011) (in Chinese with English abstract).
147.Liu, P., Jiang, Z.W., Zhang, Y.M. & Chen, Y.W. The investigation on separate furrow irrigation of film maize in southern Ningxia hill region. R*esearch of Soil and Water Conservation* **13**, 104-106 (2006) (in Chinese with English abstract).
148.Liu, X.J., Liu, P., Zhai, R.W. & Jiang, Z.W. Southern Ningxia rainfed agricultural regions maize growth stages of soil moisture content and control threshold research. *Journal of Irrigation and Drainage* **32**, 1-4 (2013) (in Chinese with English abstract).
149.Du, Y.B. Study on the technology of water-saving and increasing yield of Maize with rainwater harvesting irrigation in the level terrace of Pengyang County, Ningxia. *Ningxia Journal of Agricultural and Forest Science and Technology*, 33-33 (2010) (in Chinese with English abstract).
150.Liu, X.J., Wang, L., Zhang, H.L. Lv, Y. & Zhou, L.H. Study on the corn limited irrigation schedule at pumping areas in Yellow River of Ningxia. *Journal of Water Resources & Water Engineering* **23**, 34-37 (2012) (in Chinese with English abstract).
151.Feng, Y.X., Chen, B.D., Wang, S.L. & Cui, Y.L. Effect of different amount of nitrogen fertilizer and rainwater collecting irrigation on the yield of dryland film corn. *Gansu Agricultural Science and Technology* (1999) (in Chinese with English abstract).
152.Wang, G.Y. Relationship of temperature with grain yield and water use efficiency and nitrogen recovery efficiency in various genotypes of maize. *Dissertation for the Master Degree, Gansu Agricultural University* (2008) (in Chinese with English abstract).
153.Gao, Y.H. Research on regulation mechanism of physiological and ecological of water use efficiency of maize with plastic-film mulching. *Dissertation for the Ph.D. Degree, Gansu Agricultural University* (2012) (in Chinese with English abstract).
154.Cui, M.J. study on influence of limited water supply of maize with plastic film. *Gansu Agricultural Science and Technology*, 20-22 (2000) (in Chinese with English abstract).
155.Zhang, J. Research of water adaptive plant with maize and water use efficiency. *Dissertation for the Master Degree, Shanxi Agricultural University* (2008) (in Chinese with English abstract).
156.Bai, X.L. Experimental research on sprinkler irrigation technical modal of maize on Ordos Plateau--a case study of Otog Banner. *Dissertation for the Master Degree, Inner Mongolia Normal University* (2011) (in Chinese with English abstract).
157.Bai, G.S., Zhou, C.Y., Du, S.N. & Ren, Z.H. Effects of polyacrylamide on water use efficiency and output value of different crops in arid and semi-arid regions.

Transactions of the Chinese Society of Agricultural Engineering **31**, 101-110 (2015) (in Chinese with English abstract).
158. Zhou, C.Y., Bai, G.S., Yu, J, & Song, R.Q. Effects of super absorbent polymer on water use efficiency and output value of different crops in different rejoins. *Journal of China Agricultural University* **20**, 66-73 (2015) (in Chinese with English abstract).
159. Li, Q., Cui, M.W. & Zhang, Y.K. Appropriate irrigation quantity research on drip irrigation under membrane of corn. *Ningxia Journal of Agricultural and Forest Science and Technology* **54**, 110-111 (2013) (in Chinese with English abstract).
160. Chen, L., Yang, X.G., Zhai, D.P., Song, N.P., Yang, M.X. & Hou, J. Effects of mulching with *Caragana* powder and plastic film on soil water and maize yield. *Ningxia Journal of Agricultural and Forest Science and Technology* **31**, 108-116 (2015) (in Chinese with English abstract).
161. Zhang, W., Zhang, D.M., Fan, X.W., Liu, E.K & Chi, B.L. Influence of different tillage methods on soil surroundings and yield of maize in dryland. *Journal of Shanxi Agricultural and Sciences* **38**, 44-47 (2010) (in Chinese with English abstract).
162. Liu, Y., Yang, S.J., Li, S.Q., Chen, X.P. & Chen, F. Growth and development of maize (zea mays l.) In response to different field water management practices: resource capture and use efficiency. *Agr Forest Meteorol* **150**, 606-613 (2010).
163. Zhang, Y.W., Liu, W.Z., Wang, J. & Yang, Y.L Effects of long-term rotation and fertilization on the growth, yield and water use of spring maize. *Bulletin of Soil and Water Conservation* **30**, 124-128 (2006) (in Chinese with English abstract).
164. Xu, H.M., Zhu, L., Liu, Y., Chen, X.P. & Li, S.Q. Nitrogen absorption and allocation of spring maize on dryland of loess plateau in different farmland water management patterns. *Scientia Agricultural Sinica* **43**, 2905-2912 (2010) (in Chinese with English abstract).
165. Kanga, S., Shib, W. & Zhangc, J. An improved water-use ef®ciency for maize grown under regulated deficit irrigation. (2000).
166. Si, H. & Zhang, Z. Researching the optimal irrigation regime in china's zhuozhang river basin. *Pol J Environ Stud* **24**, 2607-2618 (2015).
167. Zhang, B.C, Liu, Y.C., Li, F.M., Cheng, Z.Y. & Liu, P.H. A Research about water supplying through soft-pipe fixed in the soil. *Journal of Irrigation and Drainage* **23**, 56-58 (2005) (in Chinese with English abstract).
168. Ren, G.S. & Chai, Q. Spatial and temporal distribution of soil moisture and its utilization efficiency in intercropped winter wheat and corn system under limited supplementary irrigation. *Bulletin of Soil and Water Conservation* **28**, 145-149 (2008) (in Chinese with English abstract).
169. Wang, Y. Research on mechanism of improving yield of maize with spring sowing and autumn mulched in the dryland. *Gansu Agricultural Science and Technology*, 19-21 (2001) (in Chinese with English abstract).
170. Wei, S.P., Wu, Q.F. & Zhang, Z.G. Spatial and temporal variation of soil water in terraced fields under different collage measures in the steep and gully region of the Loess Hilly Region. *Bulletin of Soil and Water Conservation*, 25-27 (2008) (in

Chinese with English abstract).
171. Mai, Z.Z., Jiang, R.L., Yuan, P,C., Lv, J.W., She, P., Li, M.F., Chen, Y.B., Zhang, J.X., Yang, L.& Zhao, B.T. Study on irrigation techniques for film -mulched corn in the irrigale area in central Ningxia. *Agricultural Research in the Arid Areas* **29**, 53-58 (2011) (in Chinese with English abstract).
172. Yu, A.Z. & Chai, Q. Effects of plastic film mulching and irrigation quota on yield of corn in arid oasis irrigation area. *Acta Agronomica Sinica* **41**, 778-786 (2015) (in Chinese with English abstract).
173. Dong, D.X., Shao, M.A. & Li Y.S. Study on yield potentiality of spring corn and effect of water and fertilizer in abundant rain year in Weibei Plateau. *Bulletin of Soil and Water Conservation*, 17-21 (1993) (in Chinese with English abstract).
174. Cao, J.J. Liu, Y.Z., Li, W.X., Ji, K.P., Du, Y.Y., Wang, H.L. & Zhao, W.Y. Effects of different mulching treatments on soil moisture and temperature and growth of winter wheat and summer maize. *Chinese Agricultural Science Bulletin* **29**, 107-111 (2013) (in Chinese with English abstract).
175. Zhao, X. Effects of different film removing time on summer maize growth and yield. *Dissertation for the Master Degree, Northwest University of agriculture and Forestry* (2013) (in Chinese with English abstract).
176. Zhao, Y.G. Experimental study on nitrate movement and transformation under localized compaction and ridge fertilization. *Dissertation for the Ph.D. Degree, Northwest University of agriculture and Forestry* (2002) (in Chinese with English abstract).
177. Cao, Z.K., Effects of film and straw mulching on soil moisture, soil temperature and crop growth. *Dissertation for the Ph.D. Degree, Northwest University of agriculture and Forestry* (2015) (in Chinese with English abstract).
178. Wang, C.C. Influence of soil magnistorage compatibilizer on the water consumption characteristic and water use efficiency of crops. *Dissertation for the Master Degree, Northwest University of agriculture and Forestry* (2009) in Chinese with English abstract).
179. Zhang, H. Water and nutrient use efficiency in winter wheat/summer maize rotation system under different cultivation pattern on semi-dryland farming. *Dissertation for the Master Degree, Northwest University of agriculture and Forestry* (2010) in Chinese with English abstract).
180. Zhang, H., Zhou, J.B., Wang, C.Y., Dong, F., Zheng, X.F. & Li, S.X. Effects of cultivation pattern and nitrogen rate on crop yield and water use efficiency in winter wheat-summer maize system. *Plant Nutrition and Fertilizer Science* **16**, 1078-1085 (2010) (in Chinese with English abstract).
181. Yang, X.Y., Fang, X.K., Wu, P.T. & Ye, C.H. Study on field experiment of summer corn for low border irrigation. *Chinese Agricultural Science Bulletin* **25**, 282-286 (2009) (in Chinese with English abstract).
182. Xue, L. Effect of water and nitrogen coupling under alternating furrow irrigation and N placement on summer corn. *Dissertation for the Master Degree, Northwest University of agriculture and Forestry* (2008) (in Chinese with English abstract).
183. Zhang, Y. The experimental research on water saving and increasing yield effect of

new soil amendment. *Dissertation for the Master Degree, Northwest University of agriculture and Forestry* (2008) (in Chinese with English abstract).

184. Ma, G.S. Corn yield and water use efficiency of fertilizers control model. *Dissertation for the Master Degree, Northwest University of agriculture and Forestry* (2010) (in Chinese with English abstract).
185. Xu, H,M. Effects of different cultivation management practices on dry matter accumulation and water-nitrogen utilization efficiency of spring maize on the South Loess Plateau. *Dissertation for the Master Degree, Northwest University of agriculture and Forestry* (2010) (in Chinese with English abstract).
186. Ji, Q., Sun, H.Y., Taraqqi, A.K. & Wang X.D. Impact of different tillage practices on soil organic carbon and water use efficiency under continuous wheat-maize binary cropping system. *Chinese Journal of Applied Ecology* **25**, 1029-1035 (2014) (in Chinese with English abstract).
187. Cai, Y.K. Effect of Gravel Mulching Degree on farmland moisture and water consumption features of crops rotation system. *Dissertation for the Master Degree, Northwest University of agriculture and Forestry* (2015) (in Chinese with English abstract).
188. Zhang, T. Effect of gravel mulching degree on farmland moisture and water consumption features of crops rotation system. *Dissertation for the Master Degree, Northwest University of agriculture and Forestry* (2013) (in Chinese with English abstract).
189. Zhang, T., Wu, P.T., Zhao, X.N., Zhang, F.Y. & Wang, Z.K. Effects of ridge and furrow planting system on growth and yield of maize. *Agricultural Research in the Arid Areas* **31**, 27-30 (2013) (in Chinese with English abstract).
190. Chen, X. Effects of rainfall harvesting planting combined with waterving irrigation on photosynthetic characteristics and water use efficiency of spring corn. *Dissertation for the Master Degree, Northwest University of agriculture and Forestry* (2013) (in Chinese with English abstract).
191. Yin, M.H., Li, Y.N., Zhang, T.L., Xu, Y.B., Gu, X.B. & Wang, X.Y. Effects of different rainwater harvesting patterns on soil hydrothermal regimes and water use efficiency of summer maize. *Journal of agricultural machinery* **46**, 194-203 (2015) (in Chinese with English abstract).
192. Zhang, F.Y. Experimental study on crop water regulation of ridge-furrow irrigated intercropping system. *Dissertation for the Ph.D. Degree, Chinese Academy of Agricultural Sciences* (2013) (in Chinese with English abstract).
193. Chen, G.P. Effects of alternative irrigation on yield and water use efficiency of wheat corn inter-cropping systems in Oasis area. *Dissertation for the Master Degree, Gansu Agricultural University* (2007) (in Chinese with English abstract).
194. Meng, Y., Cai, H.J. Wang, J. & Zhang, X.P. Effect of straw mulching on the growth of summer maize and soil water utilization. *Journal of Northwest Sci-Tech University of Agriculture and Forestry* **33**, 131-135 (2005) (in Chinese with English abstract).
195. Han, K. et al. Management of furrow irrigation and nitrogen application on summer maize. *Agron J* **106**, 1402-1410 (2014).

196. Han, K. et al. Separating nitrogen fertilizer and irrigation water application in an alternating furrow irrigation system for maize production. *Nutr Cycl Agroecosys* **96**, 107-122 (2013).
197. Wang, Q.M., Huo, Z.L., Zhang, L.D., Wang, J.H. & Zhao, Y. Impact of saline water irrigation on water use efficiency and soil salt accumulation for spring maize in arid regions of china. *Agr Water Manage* **163**, 125-138 (2016).
198. Liang, J.B., Liu, J.H. Yang, T. Effect of different cultivation ways to growth of maize root system and soil water content. *Journal of Anhui Agriculture Sciences* **34**, 2353-2354 (2006) (in Chinese with English abstract).
199. Sheng, Y. The Law of soil water movement and its influence on growth of crop in Oasis farmland. *Dissertation for the Master Degree, Xinjiang Agricultural University* (2004) (in Chinese with English abstract).
200. Liu, J., Zhang, H.J. & An, F.H. Study on effect of limited irrigation on soil moisture use and grain yield of spring maize in sandy land. *Agricultural Research in the Arid Areas* **29**, 7-11 (2011) (in Chinese with English abstract).
201. Su, D.R. Study on fundamentals of water saving and efficient irrigation for intercropping in arid regions. *Dissertation for the Ph.D. Degree, Chinese Academy of Agricultural Sciences* (2001) (in Chinese with English abstract).
202. Yu, B.J., Shi, P.Z., Yang, X.Y., Li, X.L. & Kang, S.Z. Study on water requirement amounts and of maize under controlled alternate irrigation in arid area. *Gansu Water Resources and Hydropower Technology* **42**, 209-212 (2006) (in Chinese with English abstract).
203. Wang, F.X. Experimental study on sowing with water of absolved water-storing irrigation technology on maize irrigation district of Hexi. *Dissertation for the Master Degree, Gansu Agricultural University* (2009) (in Chinese with English abstract).
204. Ding, L., Jin, Y.Z., Li, Y.H. & Wang, Y.B. Spatial pattern and water-saving mechanism of wheat and maize under the condition of strip-ridge intercropping. *Acta Agriculturae Boreali-occidentalis Sinica* **23**, 56-63 (2014) (in Chinese with English abstract).
205. Wang, F.X. & Ding, L. Study on the planting model of Maize under mulch drip irrigation in the Shiyang River Basin. *Gansu Water Resources and Hydropower Technology* **51**, 29-32 (2006) (in Chinese with English abstract).
206. Zhang, G.Q. Water requirement regulation and yield of Xinjiang spring maize with high yield of (≥15000 kg/hm2) under the condition of drip irrigation. *Dissertation for the Master Degree, Shihezi University* (2015) (in Chinese with English abstract).
207. Chai, Q., Yang, C.H & Huang, G.B. Characteristics of crop water consumption of different cropping in an arid oasis. *Journal of Desert Research* **30**, 1153-1159 (2010) (in Chinese with English abstract).
208. Kong, X.F., Feng, F.X. & Chai, Q. The effect of different root partition patterns on soil water condition of wheat-corn intercropping system. *Journal of Desert Research* **34**, 780-785 (2014) (in Chinese with English abstract).
209. Yang, W. Analysis of soil water characteristics of farmland and under different storage irrigation in Hexi region of Gansu province. *Dissertation for the Master*

Degree, Northwest University of agriculture and Forestry (2009) (in Chinese with English abstract).

210. Ren, L.W., Wang, X.T, Liu, M.C., Ding, W.K. & Jiang, J.F. Effects of drought stress on soil moisture dynamics and water use efficiency in corn. *Chinese Agricultural Science Bulletin* **31**, 142-147 (2015) (in Chinese with English abstract).
211. Qin, Y.Z. Effect of root-canopy interaction on water use efficiency of wheat/maize intercropping. *Dissertation for the Master Degree, Gansu Agricultural University* (2015) (in Chinese with English abstract).
212. Chen, G.P. & Yu, A.Z. Response of water use characteristics of maize/pea intercropping to different root partition and irrigation Quota. *Acta Agriculturae Boreali-occidentalis Sinica* **23**, 68-73 (2014) (in Chinese with English abstract).
213. Dai, J. Effect of water and interactions between species with root characteristics and water application in maize and pea. *Dissertation for the Master Degree, Gansu Agricultural University* (2010) (in Chinese with English abstract).
214. Li, Y., Zhao, F.N., Ding, W.Z., Ren, L.W. & Wang, H.L. Effect of irrigation modes and sowing date on maize water dynamics and water use efficiency. *Chinese Agricultural Science Bulletin* **31**, 62-67 (2015) (in Chinese with English abstract).
215. Qi, D.L., Hu, T.T., Wu, X. & Cheng, D.L. Effects of irrigation methods on root growth, yield and water use of maize. *Acta Agriculturae Boreali-occidentalis Sinica* **23**, 73-78 (2014) (in Chinese with English abstract).
216. Wang, S.G., Wang, Y.B., Ding, B. & Zhao, L. Experimental study on water storage irrigation in spring wheat and maize in Huangyang irrigation area. *Gansu Water Resources and Hydropower Technology* **49**, 14-17 (2013) (in Chinese with English abstract).
217. Wu, Y.D., Huang, G.B., Chai, Q., Yu, A.Z. & Qin, A.Z. Effect of intercropped wheat stubble retention patterns on yield and WUE of intercropped corn. *Journal of Irrigation and Drainage* **32**, 107-110 (2013) (in Chinese with English abstract).
218. Yang, C.Y. Research on water use characteristics and mechanism of alternative irrigated intercropping systems. *Dissertation for the Ph.D. Degree, Gansu Agricultural University* (2010) (in Chinese with English abstract).
219. Yu, H.L. Effects of nitrogen supply levels and wheat intercropping maize on soil carbon and nitrogen of straw mulching farmland in Hexi Oasis irrigation area. *Dissertation for the Master Degree, Gansu Agricultural University* (2012) (in Chinese with English abstract).
220. Zhang, Z.P. Integrated evaluation on water-saving cropping system in irrigated Oasis of Hexi Corridor. *Dissertation for the Master Degree, Gansu Agricultural University* (2010) (in Chinese with English abstract).
221. Li, H.Y., Zhang, R. & Wang, F.X. Effects of water-retaining agent on soil water movement and water use efficiency of maize sowed with absolved water-storing irrigation. *Transactions of the Chinese Society of Agricultural Engineering* **27**, 37-42 (2011) (in Chinese with English abstract).
222. Wu, D., Meng, W.C., Bao, X.D., Tong, L. & Du, T.S. Effect of different surface irrigation methods on yield and water use efficiency of maize for seed. *Journal of Irrigation and Drainage* **33**, 185-188 (2014) (in Chinese with English abstract).

223.Liu, Y.J., Li, Y.N., Pan, T, Zhai, L.X. & Du, Z.L. Study on effects of different irrigation treatments on evapotranspiration and yield in spring maize. *Agricultural Research in the Arid Areas* **27**, 67-72 (2009) (in Chinese with English abstract).
224.Huang, T., Hu, Z.Q., Bao, X.G., Che, Z.X. & E, S.Z. The effect of different water-saving cultivation models on crop yields and water consumption in Shiyang River area. *China Water Resources and Hydropower Technology*, 9-12 (2012) (in Chinese with English abstract).
225.Xue, F.D., Zhang, F.C., Suo, Y.S. & Zhang, P. Effect of water deficit at different growth stages on growth, yield and water use of spring maize in Hexi area. *Journal of Northwest Sci-Tech University of Agriculture and Forestry* **41**, 59-65 (2013) (in Chinese with English abstract).
226.Shi, Z.X., Chai, Q., Yang, C.X. & Qin, A.Z. Effects of different nitrogen applications and intercropping stripe compound on yield and WUE under maize / pea intercropping. *Journal of Gansu Agricultural University 46, 39-43* (2011) (in Chinese with English abstract).
227.Teng, Y.Y., Zhao, C., Chai, Q., Hu, F.L. & Feng, F.L. Effects of postponing nitrogen topdressing on water use characteristics of maize-pea intercropping system. *Acta Agriculturae Boreali Sinica* **42**, 446-455 (2016) (in Chinese with English abstract).
228.Wang, H., Jin, X.J., Tang, W.W., Zhang, X.D. &Xi, X.D. Effects of combination with compost and chemical fertilizer for maize growth and yield in Gansu inland irrigation area. *Soil and Fertilizer Sciences in China* (2012) (in Chinese with English abstract).
229.Wang, K., Zhang, H.J. & Luo, X.H. The effects of different cultivation modes on maize yield and soil water under deficit irrigation. *Journal of Irrigation and Drainage* **32**, 83-86 (2013) (in Chinese with English abstract).
230.Chen, G.D., Mechanism of water competition and niche differentiation between intercropped wheat (*Triticum aestivum*) and maize (*Zea mays* L). *Dissertation for the Ph.D. Degree, Gansu Agricultural University* (2015) (in Chinese with English abstract).
231.Yu, A.Z. Effects of plastic mulching and irrigation on soil thermal characteristics and WUE of corn. *Dissertation for the Ph.D. Degree, Gansu Agricultural University* (2013) (in Chinese with English abstract).
232.Qi, D.L. Effect of localized supply of water and nitrogen on and temporal root distribution for Maize. *Dissertation for the Master Degree, Northwest University of agriculture and Forestry* (2013) (in Chinese with English abstract).
233.Liu, S.M. Effects of spatial distribution on water and nitrogen use efficiency of pea/maize. *Dissertation for the Master Degree, Gansu Agricultural University* (2013) (in Chinese with English abstract).
234.Hu, Z.Q., Ma, Z.M., Bao, X.G. & Zhang, J.D. Effect of RDI on yield and water consumption of major crops in Shiyang River area. *Water-Saving Irrigation*, 10-13 (2010) (in Chinese with English abstract).
235.Liu, C.W., Wang, Q., Liu, Q.L., Guan, X.K., Yang, Y. & Zhang, E.H. Effects of stubble-standing mode on the grain yield and water use efficiency of wheat and maize in wheat/maize intercropping system. *Chinese Journal of Applied Ecology*

24, 438-444 (2013) (in Chinese with English abstract).
236. Luo, Z.X. Water use characteristics and influence factors of alternative irrigated wheat corn intercropping in Oasis Area. *Dissertation for the Master Degree, Gansu Agricultural University* (2008) (in Chinese with English abstract).
237. Liu, H.L. Characterization of evapotranspiration rules and their influencing factors in wheat-corn intercropping field in Oasis area. *Dissertation for the Master Degree, Gansu Agricultural University* (2009) (in Chinese with English abstract).
238. Qi, W.M. Water consumption characteristics and root distribution of wheat corn intercropping systems in Oasis Area. *Dissertation for the Master Degree, Gansu Agricultural University* (2009) (in Chinese with English abstract).
239. Shi, Z.X. Water and fertilizer use efficiency response of maize/pea intercropping to planting structure and nitrogen application rate in oasis irrigation area. *Dissertation for the Master Degree, Gansu Agricultural University* (2010) (in Chinese with English abstract).
240. Liu, Y.J. Study on irrigation schedule of corn under film hole irrigation. *Dissertation for the Master Degree, Northwest University of agriculture and Forestry* (2006) (in Chinese with English abstract).
241. Zhai, Z.F. Study on deficit irrigation schedule for maize under agronomic management *Dissertation for the Master Degree, Gansu Agricultural University* (2009) (in Chinese with English abstract).
242. Huang, P., Hou, S.J. & Chen, X.W. Effect on water, fertilizer use efficiency of corn under mineral organic fertilizer and chemical fertilizer reduction. *Chinese Agricultural Science Bulletin* **27**, 295-298 (2011) (in Chinese with English abstract).
243. Huang, P., He, T. & Du, J. Effect on water, fertilizer and light use efficiency of maize under biological bacterial fertilizer and chemical fertilizer reduction. *Chinese Agricultural Science Bulletin* **27**, 76-79 (2011) (in Chinese with English abstract).
244. Gao, F., Cui, Z.T., Liu, J., Zhang, Z.C., Han, M. & Yuan, Z.X. Effect of ridging way on yield and water use efficiency for ridge with plastic film mulching-furrow irrigation of corn. *Gansu Agricultural Science and Technology*, 12-24 (2012) (in Chinese with English abstract).
245. Yang, X.Y., Du, T.S., Pan, Y.H. & Zhang, H.R. Scheduling irrigation for maize under different irrigation methods in Minqin Oasis. *Journal of Irrigation and Drainage* **22**, 22-24 (2013) (in Chinese with English abstract).
246. Tao, Z.Q. Water use characteristics and the influence factors of wheat corn intercropping with reduced tillage and straw mulching. *Dissertation for the Master Degree, Gansu Agricultural University* (2010) (in Chinese with English abstract).
247. Wu, J.J. & Chai, Q. Characteristics of water consumption of wheat/maize intercropping system with reduced tillage and straw mulching. *Agricultural Research in the Arid Areas*, 122-127 (2014) (in Chinese with English abstract).
248. Hu, F.L., Chai, Q., Gan, Y.T., Yin, W. Zhao, C. & Feng, F.X. Characteristics of soil carbon emission and water utilization in wheat/maize intercropping with minimal/zero tillage and straw retention. *Scientia Agricultural Sinica* **49**, 120-131

(2015) (in Chinese with English abstract).
249. Wu, K.S. Effects of nitrogen fertilizer application and rhizobia inoculation on crop yields, water and nutrient utilization in pea/maize intercropping systems. *Dissertation for the Ph.D. Degree, Gansu Agricultural University* (2015) (in Chinese with English abstract).
250. Liu, C.W. Effect of stubble and tillage management on soil carbon and nitrate under wheat/maize intercropping in Oases of the Shiyanghe River Basin. *Dissertation for the Ph.D. Degree, Gansu Agricultural University* (2012) (in Chinese with English abstract).
251. Hu, T.M., Wang, Z.L. & Dong, P.G. Effect of different irrigation system of seeding maize on soil moisture and in northwest arid areas. *Water-Saving Irrigation*, 27-31 (2014) (in Chinese with English abstract).
252. Tian, Y.F. Effect of limited irrigation and nitrogen rate on spring maize growth and transfer of water and nitrogen of root zone soil in Shiyang. *Dissertation for the Master Degree, Northwest University of agriculture and Forestry* (2008) (in Chinese with English abstract).
253. Wu, Y.D. Correlation research of corn plant carbon accumulation and water consumption. *Dissertation for the Master Degree, Gansu Agricultural University* (2013) (in Chinese with English abstract).
254. Han, W.H. Wang, Z.L. & Zhang, P. Experimental study on water and nutrient schedule of seed maize with ridges planting under furrow irrigation in Shingyanghe basin. *Journal of Irrigation and Drainage* **33**, 107-111 (2014) (in Chinese with English abstract).
255. Mao, L.L. et al. Yield advantage and water saving in maize/pea intercrop. *Field Crop Res* **138**, 11-20 (2012).
256. Mu, Y. et al. Performance of wheat/maize intercropping is a function of belowground interspecies interactions. *Crop Sci* **53**, 2186-2194 (2013).
257. Yang, C., Huang, G., Chai, Q. & Luo, Z. Water use and yield of wheat/maize intercropping under alternate irrigation in the oasis field of northwest china. *Field Crop Res* **124**, 426-432 (2011).
258. Chen, G.D. et al. Belowground interspecies interaction enhances productivity and water use efficiency in maize-pea intercropping systems. *Crop Sci* **55**, 420-428 (2015).
259. Hu, F. et al. Less carbon emissions of wheat-maize intercropping under reduced tillage in arid areas. *Agron Sustain Dev* **35**, 701-711 (2015).
260. Kang, S.Z., Liang, Z.S., Pan, Y.H., Shi, P.Z. & Zhang, J.H. Alternate furrow irrigation for maize production in an arid area. *Agr Water Manage* **45**, 267-274 (2000).
261. Feng, Y.J. Xu, L.P. & Zhang, F.R. Comparative study of water law and the economic benefit of different specifications. *Gansu Water Resources and Hydropower Technology* **51**, 37-39 (2015) (in Chinese with English abstract).
262. Cao, S.Y. Study on the water saving irrigation of micro strip ridge and furrow irrigation in Hexi. *Gansu Agricultural Science and Technology*, 23-24 (2002) (in Chinese with English abstract).

263. Liang, C.Y., Ma, Z.M. & Cao, S.Y. Research on the effect of water consumption and the yield of maize on limited water supply in oasis irrigation area. *China Water Resources and Hydropower Technology* 55-57 (2013) (in Chinese with English abstract).
264. Zhang, F.Y., Wu, P.T., Zhao, X.N., Zhang, E.H. & Cheng, X.F. Effects of conservation tillage on soil water regimes and water use efficiency in farmland of Heihe river basin in northwest china. *African Journal of Agricultural Research* **6**, 5959-5966 (2011).
265. Zhang, W.Y. The Hexi oasis irrigation stubble mulching no-till planting spring corn soil ecological effect research. *Dissertation for the Master Degree, Gansu Agricultural University* (2006) (in Chinese with English abstract).
266. Zhang, Y. L., Xiao, R. Cheng, Z.Y. Effects on WUE and yield of maize with hole irrigation in Hexi Oasis irrigation region. *Water-Saving Irrigation*, 55-57 (2013) (in Chinese with English abstract).
267. Zhang, W.Y. Effect of hole irrigation on irrigation and yield of spring wheat and maize in the inland irrigation district of Hexi. *Dissertation for the Master Degree, Gansu Agricultural University* (2006) (in Chinese with English abstract).
268. Guan, W.W. The physiological mechanism of high water and nitrogen use efficiency and high-yielding for wheat intercropping maize in Hetao irrigation. *Dissertation for the Master Degree, Inner Mongolia Normal University* (2013) (in Chinese with English abstract).
269. Han, K.M. Water-saving and high-yielding irrigation regime of wheat intercropping maize and its physiological machining in Hetao irrigation District. *Dissertation for the Master Degree, Inner Mongolia Normal University* (2011) (in Chinese with English abstract).
270. Yi, W.D., Han, K.M., Zhang, Y.P. & Guan, W.W. Evaluating and selecting water saving and drought resistance varieties of corn in Hetao irrigation district. *Journal of Inner Mongolia Agricultural University* **33**, 61-67 (2012) (in Chinese with English abstract).
271. Zhang, R., Yu, J., Geng, G.J., Li, J.J. & Bai, G.S. Effects of PAM with different application methods on soil moisture, soil temperature and growth of *Zea mays*. *Science of Soil and Water Conservation* **11**, 96-103 (2013) (in Chinese with English abstract).
272. Du, S.N., Geng, G.J., Yu, J., Zhang, P.Q. & Bai, G.S. Effects of super absorbent applied by different methods on soil moisture, soil temperature and maize growth. *Bulletin of Soil and Water Conservation* **32**, 270-276 (2012) (in Chinese with English abstract).
273. Zhang, Z.W., Shi, H.B., Li, Z., Liu, D.P., Yan, J.W., Peng, Z.Y. & Ni, D.N. Effects of irrigation treatments on water use efficiency and yield in the three-dimensional planting model. *Journal of Irrigation and Drainage* **33**, 26-30 (2014) (in Chinese with English abstract).
274. Ai, H.J., Li, Z.X. Bian, L.J. Effects of spring wheat/maize spring wheat sunflower intercropping on soil moisture. *Bulletin of Soil and Water Conservation* **34**, 91-98 (2014) (in Chinese with English abstract).

275. Liu, D.P., Yang, S.Q., Shi, H.B., Du, X.F., Sun, L.Y., Chang, C.L. & Li, Z. Crop yield and water-fertilizer utilization efficiency under combined application of nitrogen and phosphorous. *Chinese Journal of Ecology* **33**, 902-909 (2014) (in Chinese with English abstract).
276. Yan, J.W. Study on the water and nitrogen migration regularity and efficient utilization of maize in the salinity soil. *Dissertation for the Ph.D. Degree, Inner Mongolia Normal University* (2014) (in Chinese with English abstract).
277. Fan, Y. J. Study on Corn and sunflower optimize irrigation schedules of drip irrigation under film in Hetao irrigation district. *Dissertation for the Master Degree, Inner Mongolia Normal University* (2014) (in Chinese with English abstract).
278. Gong, X.W. Characteristics of soil temperature and soil water for inter-planting crop under mulched drip irrigation in arid areas. *Dissertation for the Master Degree, Inner Mongolia Normal University* (2013) (in Chinese with English abstract).
279. Peng, Z.Y. Study on water consumption and irrigation schedule in an intercropping field under mulched drip irrigation. *Dissertation for the Master Degree, Inner Mongolia Normal University* (2015) (in Chinese with English abstract).
280. Lv, Y.J. The experimental study of influence of biochar fertilizer on soil properties, maize growth and water and fertilizer use efficiency. *Dissertation for the Master Degree, Inner Mongolia Normal University* (2014) (in Chinese with English abstract).
281. Ren, W.J. & Li, Y.H. Efficiency of water application of Maize rainwater irrigation in semi-arid corn area. *Modern Agricultural Sciences*, 43-45 (2010) (in Chinese with English abstract).
282. Li, X.L., Liu, J.H. & Cheng, Z.Y. Experimental research on effect of water-saving and yield increase for maize under different irrigation techniques. *Water-Saving Irrigation*, 7-9 (2006) (in Chinese with English abstract).
283. Li, W.M. Influences of different film mulching modes in spring Maize physiological characteristics and soil environment under drip irrigation. *Dissertation for the Master Degree, Inner Mongolia Normal University* (2014) (in Chinese with English abstract).
284. Luo, D.H., Wang, Y. & Yu, Z. Influence of drip irrigation quota on film maize growth, yield and water use efficiency. *Water-Saving Irrigation*, 5-8 (2015) (in Chinese with English abstract).
285. Lv, D.M. Effects of different moisture treatments on corn growth, water consumption and of maize. *Water-Saving Irrigation*, 40-43 (2015) (in Chinese with English abstract).
286. Li, X.Y. & Gong, J.D. Effects of different ridge: furrow ratios and supplemental irrigation on crop production in ridge and furrow rainfall harvesting system with mulches. *Agr Water Manage* **54**, 243-254 (2002).
287. Wang, Q. Yang, J.Q. Study on drip irrigation program of winter wheat and crop in Ningxia yellow river irrigation district. *Journal of Anhui Agriculture Sciences* **40**, 17585-17588 (2012) (in Chinese with English abstract).
288. Hou, Z., Du, J. & Shen, Z.R. Study on key technology of drip fertilization for corn in Ningxia Yellow River irrigation district. *Water-Saving Irrigation*, 9-12 (2012)

(in Chinese with English abstract).
289. Gao, J.L., Wang, Q.G., Sang, D.D., Wang, J.X., Wang, J. & Chang, C.X. The space-time dynamics of soil water and the mechanism of high water use efficiency of covering film among furrows on spring maize. *Journal of Maize Sciences* **16**, 39-45 (2008) (in Chinese with English abstract).
290. Wu, L.B. Basic study on water-saving and high yield cultivation physiology of spring maize by agronomy adjust. *Dissertation for the Master Degree, Inner Mongolia Normal University* (2007) (in Chinese with English abstract).
291. Sang, D.D. Leaf senescence of spring maize super high yield population and water saving regulation. *Dissertation for the Master Degree, Inner Mongolia Normal University* (2009) (in Chinese with English abstract).
292. Zhang, C.W., Jiang, X.F., Zhang, G.P., Guo, T.W. & Chen, N.L. Effects of macromolecule polymers on the water use efficiency and yield of corn in sandy soil. *Gansu Agricultural Science and Technology*, 49-524 (2015) (in Chinese with English abstract).
293. Yu, Y.X., Li, J.R. & Yao. X.Z. The effect of different cropping patterns and irrigation amounts on maize yield in Jingtai irrigated area. *Modern Agricultural Sciences*, 16-17 (2013) (in Chinese with English abstract).
294. Zhang, T.H. & Zhao, X.Y. Effect of various amounts of NP fertilizers on yield of spring corn at sandy cropland. *Journal of Desert Research* **30**, 1153-1159 (1999) (in Chinese with English abstract).
295. Shi, Q.Q. Coupling Effects of reduced tillage and straw covering on soil carbon emission in wheat-maize intercropping system. *Dissertation for the Master Degree, Gansu Agricultural University* (2015) (in Chinese with English abstract).
296. Liu, Y. Study of the effects of limited water supply on corn covered with membrane on dryland. R*esearch of Soil and Water Conservation* **10**, 101-103 (2003) (in Chinese with English abstract).
297. Yu, Y. The experimental study on coupling effect mechanism and model of crop response to water of un- sufficient irrigation under intercropping condition. *Dissertation for the Master Degree, Inner Mongolia Normal University* (2010) (in Chinese with English abstract).
298. Wang, Z.K., Zhao, X.N., Wu, P.T. & Chen, X.L. Effects of water limitation on yield advantage and water use in wheat (triticum aestivum l.)/Maize (zea mays l.) Strip intercropping. *Eur J Agron* **71**, 149-159 (2015).
299. Wang, Z.K., Wu, P.T., Zhao, X.N., Gao, Y. & Chen, X.L. Water use and crop coefficient of the wheat-maize strip intercropping system for an arid region in northwestern china. *Agr Water Manage* **161**, 77-85 (2015).
300. Zhao, N., Huang, X.F., Ren, X.N., Yang, J.G., Zhang, X.H., Liu, X.H. & Ji, L.D. Water requirement characteristics of spring corn under mulched drip irrigation in the Yellow River irrigation region of Ningxia. *Journal of Irrigation and Drainage* **33**, 31-34 (2014) (in Chinese with English abstract).
301. Han, B.F., Tian, J.C. & Yang, J.Z. Effects of film hole irrigation on change characteristics of soil water and salt in the maize field. *Journal of Soil and Water Conservation* **29**, 252-257 (2015) (in Chinese with English abstract).

302. Yao, J.Q. The effect of different saving water technology on the maize water use efficiency. *Journal of Inner Mongolia Agricultural University*, 34-35 (2008) (in Chinese with English abstract).
303. Liu, J.Z. Farmland protection cultivation experimental study in the interlocks area of the farming and animal husbandry of Jingbian. *Dissertation for the Master Degree, Northwest University of agriculture and Forestry* (2006) (in Chinese with English abstract).
304. Ji, X.L., Zhang, X. & Liu, C.Y. Study on water saving technology of corn in northern Fengshatan agronomic irrigation area of Yulin. *Shanxi Agricultural Science* **59**, 27-31 (2013) (in Chinese with English abstract).
305. Wang, W. & Zhang, X. Effects of different irrigation mechods on growth and yields of spring maize in sandy area of Yulin city. *Bulletin of Soil and Water Conservation* **35**, 213-217 (2015) (in Chinese with English abstract).
306. Zheng, Y.J. Analysis and verification on technological model of high yield and high efficiency in maize. *Dissertation for the Master Degree, Northwest University of agriculture and Forestry* (2012) (in Chinese with English abstract).
307. Zhang, K.K. & Zheng, L.L. The effect of mulching and supplemental irrigation on the soil humidity of maize in the arid regions of Gansu. *Water-Saving Irrigation*, 51-54 (2016) (in Chinese with English abstract).
308. Li, X., Shi, H.B. Cheng, M.J., Ma, L.Z & Li, B. Effects of the supplemental irrigation of harvested rainwater. *Transactions of the Chinese Society of Agricultural Engineering* **23**, 34-38 (2007) (in Chinese with English abstract).
309. Shen, Q.Y., Tian, J.Q., Wang, Y.H., Tao, W.X. & Wang, J.L. Study on water use efficiency of monoculture maize under various cultivated way in yellow river irrigation district of Ningxia municipality. *Water-Saving Irrigation*, 3-6 (2004) (in Chinese with English abstract).
310. Ma, W.L. Study on farming system and planting structure for saving-water in Yellow River irrigation area of Ningxia. *Dissertation for the Master Degree, Ningxia Agriculture University* (2005) (in Chinese with English abstract).
311. Feng, H.L. The effect of regulated deficit irrigation on growth-development characteristics and water use efficiency of maize. *China Rural Water and Hydropower* **50**, 10-13 (2016) (in Chinese with English abstract).
312. An, F.H. Study on soil water and yield of spring maize under limited irrigation. *Dissertation for the Master Degree, Gansu Agricultural University* (2009) (in Chinese with English abstract).
313. Fan, Y.B., Wang, C.G. & Nan, Z.B. Comparative evaluation of crop water use efficiency, economic analysis and net household profit simulation in arid northwest china. *Agr Water Manage* **146**, 335-345 (2014).
314. Wang, N. The influence of physiological characteristics and water use efficiency for different crop varieties under salt stress. *Dissertation for the Master Degree, Inner Mongolia Normal University* (2012) (in Chinese with English abstract).
315. Lv, J.L. Effect on water consumption and water use efficiency of high yield spring maize under tillage and plastic film mulching. *Dissertation for the Master Degree, Inner Mongolia Normal University* (2013) (in Chinese with English abstract).

316.Zhang, H., Hao,C.L., Meng, F.S., Ci, Y.H., Zheng, W., Zhang, L.Y. ,Bian, L.M., Dong,Z. & Huo, J.F. Effects of different irrigation quota on yield and soil moisture of maize with drip irrigation under film mulch. *Crops*, 105-109 (2016) (in Chinese with English abstract).
317.Yuan, J.J., Li, G.Y. & Yuan, Z.C. Effects of mulched drip irrigation and subsurface drip irrigation on corn water consumption growth and yield. *Journal of Irrigation and Drainage* **34**, 93-98 (2015) (in Chinese with English abstract).
318.Xu, Z.Q., Zhang, F.J., Gao, J.L., & Hu, S.P. The study of maize water use efficiency and growth characteristics under drip irrigation. *Journal of Inner Mongolia Agricultural University* **43**, 1-5 (2015) (in Chinese with English abstract).
319.Zhou, L. Study of water-saving irrigation on corn in inner Mogolia Alashan left banner desert oasis agriculture area. *Dissertation for the Master Degree, Ningxia Agriculture University* (2014) (in Chinese with English abstract).
320.Hou, Q. & Shen, J.G. A study on optimal irrigation index for spring wheat and maize in major irrigation area of Inner Mongolia. *Agricultural Research in the Arid Areas* **19**, (2001) (in Chinese with English abstract).
321.Ning, D.N., Li, R.P., Shi, H.B., Miao, Q.S. & Li, Z. Effects of different irrigation methods on transport of root zone soil water-salt and yield of maize under inter-planting mode. *Soils* **43**, 1797-804 (2015) (in Chinese with English abstract).
322.Aydinsakir, K., Erdal, S., Buyuktas, D., Bastug, R. & Toker, R. The influence of regular deficit irrigation applications on water use, yield, and quality components of two corn (*Zea Mays* L.) Genotypes. *Agr Water Manage* **128**, 65-71 (2013).
323.Dagdelen, N., Yilmaz, E., Sezgin, F. & Gurbuz, T. Water-yield relation and water use efficiency of cotton (gossypium hirsutum l.) And second crop corn (*Zea Mays* L.) In western turkey. *Agr Water Manage* **82**, 63-85 (2006).
324.Ibragimov, N. et al. Permanent beds vs. Conventional tillage in irrigated arid central asia. *Agron J* **103**, 1002-1011 (2011).
325.Kirda, C. et al. Grain yield response and N-fertiliser recovery of maize under deficit irrigation. *Field Crop Res* **93**, 132-141 (2005).
326.Kiziloglu, F.M., Sahin, U., Kuslu, Y. & Tunc, T. Determining water-yield relationship, water use efficiency, crop and pan coefficients for silage maize in a semiarid region. *Irrigation Sci* **27**, 129-137 (2009).
327.Yazar, A., Sezen, S.M. & Gencel, B. Drip irrigation of corn in the southeast Anatolia project (gap) area in turkey. *Irrigation & Drainage* **51**, 293-300 (2002).
328.Bozkurt, Y., Yazar, A., Gencel, B. & Sezen, M.S. Optimum lateral spacing for drip-irrigated corn in the mediterranean region of turkey. *Agr Water Manage* **85**, 113-120 (2006).
329.Sepaskhah, A.R. & Khajehabdollahi, M.H. Alternate furrow irrigation with different irrigation intervals for maize (zea mays l.). *Plant Prod Sci* **8**, 592-600 (2005).
330.Feyzbakhsh, M.T., Kamkar, B., Mokhtarpour, H. & Asadi, M.E. Effect of soil water management and different sowing dates on maize yield and water use efficiency under drip irrigation system. *Arch Agron Soil Sci* **61**, 1581-1592 (2015).
331.Yilmaz, E., Akcay, S., Gurbuz, T., Dagdelen, N. & Sezgin, F. Effect of different

water stress on the yield and yield components of second crop corn in semiarid climate. *Journal of Food Agriculture & Environment* **8**, 415-421 (2010).
332. Kuscu, H., Karasu, A., Oz, M., Demir, A.O. & Turgut, I. Effect of irrigation amounts applied with drip irrigation on maize evapotranspiration, yield, water use efficiency, and net return in a sub-humid climate. *Turk J Field Crops* **18**, 13-19 (2013).
333. Sepaskhah, A.R. & Parand, A.R. Effects of alternate furrow irrigation with supplemental every-furrow irrigation at different growth stages on the yield of maize (l.). *Plant Prod Sci* **9**, 415-421 (2006).
334. Kuscu, H. & Demir, A.O. Yield and water use efficiency of maize under deficit irrigation regimes in a sub-humid climate. *Philipp Agric Sci* **96**, 32-41 (2013).
335. Simsek, M., Can, A., Denek, N. & Tonkaz, T. The effects of different irrigation regimes on yield and silage quality of corn under semi-arid conditions. *African Journal of Biotechnology* **10**, 5869-5877 (2011).
336. Oktem, A., Simsek, M. & Oktem, A.G. Deficit irrigation effects on sweet corn (*Zea Mays Saccharata Sturt*) with drip irrigation system in a semi-arid region: i. Water-yield relationship. *Agr Water Manage* **61**, 63-74 (2003).
337. Lack, S., Dashti, H., Abadooz, G. & Modhej, A. Effect of different levels of irrigation and planting pattern on grain yield, yield components and water use efficiency of corn grain (zea mays l.) Hybrid sc. 704. *African Journal of Agricultural Research* **7**, 2873-2878 (2012).
338. Mojaddam, M., Torkii, T.A. & Abadouz, G.R. Effect of irrigation amount on yield, yield components and water use efficiency of corn hybrids. *American-Eurasian Journal of Sustainable Agriculture* **6**, 1-6 (2012).
339. Bahrani, Pourreza, Madani & Amiri Effect of PRD irrigation method and potassium fertilizer application on corn yield and water use efficiency. *Bulgarian Journal of Agricultural Science* **18**, 616 (2012).
340. Azizian, A. & Sepaskhah, A.R. Maize response to water, salinity and nitrogen levels: yield-water relation, water-use efficiency and water uptake reduction function. *Int J Plant Prod* **8**, 183-214 (2014).
341. Lv, G.H., Kang, Y.H., Li, L. & Liu, S.P. Nutrient distribution, growth, and water use efficiency in maize following winter wheat irrigated by sprinklers or surface irrigation. *Irrig Drain* **60**, 338-347 (2011).
342. Adamu, C. & Kumar, A. Physiological response, molecular analysis and water use efficiency of maize (*Zea mays* L.) Hybrids grown under various irrigation regimes. *African Journal of Biotechnology* **1329**, 2966-2976 (2014).
343. Ghamarnia, H., Parandyn, M.A., Arji, I. & Rezvani, V. An evaluation and comparison of drip and conventional furrow irrigation methods on maize. *Archives of Agronomy & Soil Science* **59**, 733-751 (2013).
344. Abedinpour, M. et al. Performance evaluation of Aquacrop model for maize crop in a semi-arid environment. *Agr Water Manage* **110**, 55-66 (2012).
345. Akhtar, M., Fayyaz-ul-Hassan, Ahmed, M., Hayat, R. & Stockle, C.O. Is rainwater harvesting an option for designing sustainable cropping patterns for rainfed agriculture? *Land Degrad Dev* **27**, 630-640 (2016).

346. Hammad, H.M., Ahmad, A., Abbas, F. & Farhad, W. Optimizing water and nitrogen use for maize production under semiarid conditions. *Turkish Journal of Agriculture & Forestry* **36**, 519-532 (2012).
347. Hammad, H.M. et al. Water and nitrogen productivity of maize under semiarid environments. *Crop Sci* **55**, 877-888 (2015).
348. Iqbal, M.A., Bodner, G., Heng, L.K., Eitzinger, J. & Hassan, A. Assessing yield optimization and water reduction potential for summer-sown and spring-sown maize in Pakistan. *Agr Water Manage* **97**, 731-737 (2010).
349. Kar, G. & Verma, H.N. Phenology based irrigation scheduling and determination of crop coefficient of winter maize in rice fallow of eastern India. *Agr Water Manage* **75**, 169-183 (2005).
350. Kar, G., Verma, H.N. & Singh, R. Effects of winter crop and supplemental irrigation on crop yield, water use efficiency and profitability in rainfed rice based cropping system of eastern India. *Agr Water Manage* **79**, 280-292 (2006).
351. Sampathkumar, T., Pandian, B.J., Rangaswamy, M.V., Manickasundaram, P. & Jeyakumar, P. Influence of deficit irrigation on growth, yield and yield parameters of cotton-maize cropping sequence. *Agr Water Manage* **130**, 90-102 (2013).
352. Mishra, H.S., Rathore, T.R. & Savita, U.S. Water-use efficiency of irrigated winter maize under cool weather conditions of India. *Irrigation Sci* **21**, 27-33 (2001).
353. Sampathkumar, T., Pandian, B.J., Ranghaswamy, M.V. & Manickasundaram, P. Yield and water relations of cotton-maize cropping sequence under deficit irrigation using drip system. *Irrig Drain* **61**, 208-219 (2012).
354. Singh, B.R. & Singh, D.P. Agronomic and physiological-responses of sorghum, maize and pearl-millet to irrigation. *Field Crop Res* **42**, 57-67 (1995).
355. Amin, M.T., Anjum, L., Alazba, A.A. & Rizwan, M. Effect of the irrigation frequency and quality on yield, growth and water productivity of maize crops. *Quality Assurance & Safety of Crops & Foods* **1**, 1-10 (2015).
356. Sani, B.M., Danmowa, N.M., Sani, Y.A. & Jaliya, M.M. Growth, yield and water use efficiency of maize-sorghum intercrop at samara, northern guinea savannah, Nigeria. *Nigerian Journal of Basic & Applied Sciences* **19** (2011).
357. Rajkumara, S., Gundlur, S.S., Neelakanth, J.K. & Ashoka, P. Impact of irrigation and crop residue management on maize (*Zea Mays*)-chickpea (*Cicer Arietinum*) sequence under no tillage conditions. *Indian J Agr Sci* **84**, 43-48 (2014).
358. Prasad, U.K., Prasad, T.N., Gupta, A.K. & Prasad, S.S. Influence of irrigation on yield, profile moisture use, water-use efficiency and net return of winter maize (Zea mays) and potato (*Solanum Tuberosum*) intercropping. *Indian J Agr Sci* **67**, 193-196 (1997).
359. Karimi, M. & Gomrokchi, A. Yield and water use efficiency of corn planted in one or two rows and applying furrow or drip tape irrigation systems in Ghazvin province, Iran. *Irrigation & Drainage* **60**, 35-41 (2011).
360. Bharwana, M.A. Effect of different levels of irrigation on maize yield, water use efficiency and soil properties under different sowing methods. (2012).
361. Tesfayeteferayigezu, Narayanan, K. & Hordof, T. Effect of furrow length and flow rate on irrigation performances and yield of maize. *International Journal of*

Engineering & Technical Research **V5** (2016).
362. Shariotullah, M., Mojid, M.A., Tabriz, S.S., Acharjee, T.K. & Adham, A.K.M. Growth and yield of three hybrid maize varieties under different irrigation levels. *International Journal of Agricultural Technology* **9**, 1749-1758 (2013).
363. Kaur, B. Effect of different drip irrigation and fertigation levels on water use efficiency and yield of spring maize (Zea mays l.). (2013).
364. Shafiq, M., Hassan, I. & Hussain, Z. Maize crop production and water use efficiency as affected by planting methods. *Asian Journal of Plant Sciences*, 141-144 (2003).
365. Khan, A.G., AnwarulHassan, Iqbal, M. & Ullah, E. Assessing the performance of different irrigation techniques to enhance the water use efficiency and yield of maize under deficit water supply. *Soil & Environment* (2015).
366. Karam, F., Karaa, K. & Tarabey, N. Effects of deficit irrigation on yield and water use efficiency of some crops under semi-arid conditions of the bekah valley of Lebanon. *Water Use Efficiency & Water Productivity* (2007).
367. Istanbulluoglu, A., Kocaman, I. & Konukcu, F. Water use - production relationship of maize under Tekirdag conditions in Turkey. *Pakistan Journal of Biological Sciences* **5** (2002).
368. Alberto, M.C.R. et al. Carbon uptake and water productivity for dry-seeded rice and hybrid maize grown with overhead sprinkler irrigation. *Field Crop Res* **146**, 51-65 (2013).
369. Fandika, I.R., Kadyampakeni, D., Bottomani, C. & Kakhlwa, H. Comparative response of varied irrigated maize to organic and inorganic fertilizer application. *Phys Chem Earth* **32**, 1107-1116 (2007).
370. Fandika, I.R., Kadyampakeni, D. & Zingore, S. Performance of bucket drip irrigation powered by treadle pump on tomato and maize/bean production in malawi. *Irrigation Sci* **30**, 57-68 (2012).
371. Mavimbela, S.S.W. & van Rensburg, L.D. Integrating micro-flood irrigation with in-field rainwater harvesting: maize yield and water use efficiency. *Irrig Drain* **61**, 70-81 (2012).
372. Gobeze, Y.L. Effect of spatial arrangements of row spacing and plant density on water use and water use efficiency of maize under irrigation. (2016).
373. Kadyampakeni, D.M., Kazombophiri, S., Mati, B. & Fandika, I.R. Impacts of small-scale water management interventions on crop yield, water use and productivity in two agro-ecologies of Malawi. *Irrigation & Drainage* **05**, 215, 227 (2014).
374. Yeboah, S. Monitoring of soil moisture regime and water use efficiency under maize cowpea cropping system. **3**, 837-848 (2014).
375. El-Hendawy, S.E., Hokam, E.M. & Schmidhalter, U. Drip irrigation frequency: the effects and their interaction with nitrogen fertilization on sandy soil water distribution, maize yield and water use efficiency under Egyptian conditions. *J Agron Crop Sci* **194**, 180-192 (2008).
376. El-Hendawy, S.E. & Schmidhalter, U. Optimal coupling combinations between irrigation frequency and rate for drip-irrigated maize grown on sandy soil. *Agr*

Water Manage **97**, 439-448 (2010).
377. Pandey, R.K., Maranville, J.W. & Admou, A. Deficit irrigation and nitrogen effects on maize in a aphelian environment i. Grain yield and yield components. *Agr Water Manage* **46**, 1-13 (2000).
378. Idinoba, M.E., Idinoba, P.A., Gbadegesin, A.S. & Jagtap, S.S. Water use and seasonal differences in maize performance in the transitional humid zone of Nigeria. *Journal of Sustainable Agriculture* **24**, 37-50 (2004).
379. El Afandi, G., Khalil, F.A. & Ouda, S.A. Using irrigation scheduling to increase water productivity of wheat-maize rotation under climate change conditions. *Chil J Agr Res* **70**, 474-484 (2010).
380. Igbadun, H.E., Salim, B.A., Tarimo, A.K.P.R. & Mahoo, H.F. Effects of deficit irrigation scheduling on yields and soil water balance of irrigated maize. *Irrigation Sci* **27**, 11-23 (2008).
381. Igbadun, H.E., Tarimo, A.K.P.R., Salim, B.A. & Mahoo, H.F. Evaluation of selected crop water production functions for an irrigated maize crop. *Agr Water Manage* **94**, 1-10 (2007).
382. Abuarab, M., Mostafa, E. & Ibrahim, M. Effect of air injection under subsurface drip irrigation on yield and water use efficiency of corn in a sandy clay loam soil. *Journal of Advanced Research* **4**, 493-499 (2013).
383. Sani, B.M., Oluwasemire, K.O. & Mohammed, H.I. Effect of irrigation and plant density on the growth, yield and water use efficiency of early maize in the Nigerian savanna. *Journal of Agricultural & Biological Science* (2008).
384. El-Wahed, M.H.A. & Ali, E.A. Effect of irrigation systems, amounts of irrigation water and mulching on corn yield, water use efficiency and net profit. *Agr Water Manage* **120**, 64-71 (2013).
385. Abd El-Halim, A. Impact of alternate furrow irrigation with different irrigation intervals on yield, water use efficiency, and economic return of corn. *Chil J Agr Res* **73**, 26-27 (2013).
386. Khaledian, M.R., Mailhol, J.C., Ruelle, P. & Mubarak, I. Impacts of direct seeding into mulch on the yield, water use efficiency and nitrogen dynamics of corn, sorghum and durum wheat. *Irrigation & Drainage* **61**, 398-409 (2012).
387. Sherif, S.A., Mohamed, W.K., Ibrahim, S.T., Osman, H.E. & Elkhatib, S.I. Effect of tillage treatments and intercropping patterns on water use efficiency and yield components of soybean and maize. *Arab Universities Journal of Agricultural Sciences* (2006).
388. Mansour, A.G. Effect of localized irrigation systems and humid compost fertilizer on water and fertilizer use efficiency of maize in sandy soil. *Australian Journal of Agricultural Research* **2**, 292-297 (2013).
389. Okasha, E.M., Abdelraouf, R.E. & Abdou, M.A.A. Effect of land leveling and water applied methods on yield and irrigation water use efficiency of maize (*Zea mays* L.) Grown under clay soil conditions. *World Applied Sciences Journal* **27**, 183-190 (2013).
390. El-Tantawy, M.M., Ouda, S.A. & Khalil, F.A. Irrigation scheduling for maize grown under middle Egypt conditions. *Research Journal of Agriculture &*

Biological Sciences **3**, 456-462 (2007).

391. Mudenda, E.M., Phiri, E., Chabala, L.M. & Sichingabula, H.M. Water use efficiency of maize varieties under rain-fed conditions in zambia. *Sustainable Agriculture Research* **6**, 1 (2016).
392. Abdrabbo, M.A.A., Hassanein, M.K. & Medany, M.A. Improving water use efficiency for drip irrigation maize in Egypt. **2121**, 478-485478 (2006).
393. Ouda, S.A., Khalil, F.A. & Yousef, H. Using adaptation strategies to increase water use efficiency for maize under climate change conditions. (2013).
394. Kotb, M.A.A. & Mansour, A.A. Improving water use efficiency and yield of maize (zea mays l.) By foliar application of glycine betaine under induced water stress conditions.
395. Kubota, A. et al. Evaluation of intercropping system of maize and leguminous crops in the Nile delta of Egypt. *Japanese Journal of Tropical Agriculture* **59**, 14-19 (2015).
396. Sitoe, M.M. Improving dryland maize (zea mays l.) Water productivity in the Chokwe district of Mozambique through better nutrient management. *Water Productivity* (2013).
397. Ezekiel, B.O., Igbadun, H., Mudiare, O.J. & Oyebode, M.A. Water use efficiency of maize crop under deficit irrigation scheduling using gravity drip system in Samaru, Nigeria.
398. Ansari, H., Mirlatifi, S.M. & Farshid, A.A. Effects of deficit irrigation on yield and water use efficiency of early corn. (2006).
399. Kresovic, B. et al. Grain yield and water use efficiency of maize as influenced by different irrigation regimes through sprinkler irrigation under temperate climate. *Agr Water Manage* **169**, 34-43 (2016).
400. Kostadinov, G. & Moteva, M. Optimum use of the irrigational water in a maize (grain) field. *Trends in Agricultural Engineering 2010*, 322-328 (2010).
401. Domuţa, C. The influence of the irrigation on the water consumption, yield and on the water use efficiency in maize from the Crisurilor Plain. *Natural Resources\s&\ssustainable Development* (2009).
402. Borza, I. Researches regarding the influence of the plant density on yield and water use efficiency in the maize crop from crisurilor plain. *Analele Universităţii Din Oradea Fascicula Protecţia Mediului* (2008).
403. Moteva, M., Spalevic, V., Gigova, A. & Tanaskovik, V. Water use efficiency and yield-dependences for canola (*brassica napus*, l.) Under irrigation. *Agriculture & Forestry* (2016).
404. Afzal, M., Battilani, A., Solimando, D. & Ragab, R. Improving water resources management using different irrigation strategies and water qualities: field and modelling study. *Agr Water Manage* **176**, 40-54 (2016).
405. Amaducci, S., Colauzzi, M., Battini, F., Fracasso, A. & Perego, A. Effect of irrigation and nitrogen fertilization on the production of biogas from maize and sorghum in a water limited environment. *Eur J Agron* **76**, 54-65 (2016).
406. Di Paolo, E. & Rinaldi, M. Yield response of corn to irrigation and nitrogen fertilization in a Mediterranean environment. *Field Crop Res* **105**, 202-210 (2008).

407.Farré, I. & Faci, J.M. Comparative response of maize (*Zea Mays* L.) And sorghum (*sorghum bicolor* L. Moench) to deficit irrigation in a Mediterranean environment. *Agr Water Manage* **83**, 135-143 (2006).
408.Couto, A., Padin, A.R. & Reinoso, B. Comparative yield and water use efficiency of two maize hybrids differing in maturity under solid set sprinkler and two different lateral spacing drip irrigation systems in Leon, Spain. *Agr Water Manage* **124**, 77-84 (2013).
409.Katerji, N., Campi, P. & Mastrorilli, M. Productivity, evapotranspiration, and water use efficiency of corn and tomato crops simulated by Aquacrop under contrasting water stress conditions in the Mediterranean region. *Agr Water Manage* **130**, 14-26 (2013).
410.Salem, H.M., Valero, C., Munoz, M.A., Rodriguez, M.G. & Silva, L.L. Short-term effects of four tillage practices on soil physical properties, soil water potential, and maize yield. *Geoderma* **237**, 60-70 (2015).
411.Karam, F., Breidy, J., Stephan, C. & Rouphael, J. Evapotranspiration, yield and water use efficiency of drip irrigated corn in the Bekaa valley of Lebanon. *Agr Water Manage* **63**, 125-137 (2003).
412.Boulal, H., Gomez-Macpherson, H. & Villalobos, F.J. Permanent bed planting in irrigated mediterranean conditions: short-term effects on soil quality, crop yield and water use efficiency. *Field Crop Res* **130**, 120-127 (2012).
413.Alkaisi, M.M. & Yin, X. Effects of nitrogen rate, irrigation rate, and plant population on corn yield and water use efficiency. *Agron J* **95**, 1475-1482 (2003).
414.Manderscheid, R., Erbs, M. & Weigel, H.J. Interactive effects of free-air co2 enrichment and drought stress on maize growth. *Eur J Agron* **52**, 11-21 (2014).
415.Mastrorilli, M., Katerji, N. & Ben Nouna, B. Using the CERES-maize model in a semi-arid Mediterranean environment. Validation of three revised versions. *Eur J Agron* **19**, 125-134 (2003).
416.Fernández, J.E. et al. Water use and yield of maize with two levels of nitrogen fertilization in SW Spain. *Agr Water Manage* **29**, 215-233 (1996).
417.Farré, I. & Faci, J.M. Deficit irrigation in maize for reducing agricultural water use in a Mediterranean environment. *Agr Water Manage* **96**, 383-394 (2009).
418.O'Neill, C.J., Humphreys, E., Louis, J. & Katupitiya, A. Maize productivity in Southern New South Wales under furrow and pressurized irrigation. *Australian Journal of Experimental Agriculture* **48**, 285-295 (2008).
419.Neal, J.S., Fulkerson, W.J. & Hacker, R.B. Differences in water use efficiency among annual forages used by the dairy industry under optimum and deficit irrigation. *Agr Water Manage* **98**, 759-774 (2011).
420.Temesgen, A., Fukai, S. & Rodriguez, D. As the level of crop productivity increases: is there a role for intercropping in smallholder agriculture. *Field Crop Res* **180**, 155-166 (2015).
421.Araya, A., Kisekka, I., Gowda, P.H. & Prasad, P.V.V. Evaluation of water-limited cropping systems in a semi-arid climate using DSSAT-CSM. *Agr Syst* **150**, 86-98 (2017).
422.Hao, B. et al. Water use and grain yield in drought-tolerant corn in the Texas high

plains. *Agron J* **107**, 1922-1930 (2015).
423.Hao, B.Z. et al. Soil water extraction, water use, and grain yield by drought-tolerant maize on the texas high plains. *Agr Water Manage* **155**, 11-21 (2015).
424.Howell, T.A., Tolk, J.A., Schneider, A.D. & Evett, S.R. Evapotranspiration, yield, and water use efficiency of corn hybrids differing in maturity. **90**, 3-9 (1998).
425.Irmak, S. & Djaman, K. Effects of planting date and density on plant growth, yield, evapotranspiration, and water productivity of subsurface drip-irrigated and rainfed maize. *T Asabe* **59**, 1235-1256 (2016).
426.Irmak, S., Djaman, K. & Rudnick, D.R. Effect of full and limited irrigation amount and frequency on subsurface drip-irrigated maize evapotranspiration, yield, water use efficiency and yield response factors. *Irrigation Sci* **34**, 271-286 (2016).
427.Kapanigowda, M., Stewart, B.A., Howell, T.A., Kadasrivenkata, H. & Baumhardt, R.L. Growing maize in clumps as a strategy for marginal climatic conditions. *Field Crop Res* **118**, 115-125 (2010).
428.Stegman, E.C. Corn grain yield as influenced by timing of evapotranspiration deficits. *Irrigation Sci* **3**, 75-87 (1982).
429.Musick, J.T. & Allen, R.R. Deep ripping and blocked furrow effects on lower 1/3 furrow irrigation infiltration. *Appl Eng Agric* **17**, 41-48 (2001).
430.Payero, J.O., Tarkalson, D.D., Irmak, S., Davison, D. & Petersen, J.L. Effect of irrigation amounts applied with subsurface drip irrigation on corn evapotranspiration, yield, water use efficiency, and dry matter production in a semiarid climate. *Agr Water Manage* **95**, 895-908 (2008).
431.Yazar, A., Howell, T.A., Dusek, D.A. & Copeland, K.S. Evaluation of crop water stress index for LEPA irrigated corn. *Irrigation Sci* **18**, 171-180 (1999).
432.Suyker, A.E. & Verma, S.B. Evapotranspiration of irrigated and rainfed maize-soybean cropping systems. *Agr Forest Meteorol* **149**, 443-452 (2009).
433.Carr, T., Yang, H. & Ray, C. Temporal variations of water productivity in irrigated corn: an analysis of factors influencing yield and water use across central Nebraska. *Plos One* **11**, e161944 (2016).
434.Lyon, D.J., Boa, F. & Arkebauer, T.J. Water-yield relations of several spring-planted dryland crops following winter wheat. *Journal of Production Agriculture* **8**, 281 (1995).
435.Lamm, F.R., Trooien, T.P., Manges, H.L. & Sunderman, H.D. Nitrogen fertilization for subsurface drip-irrigated corn. *Transactions of the Asae* **533**, 533-542 (2001).
436.Steele, D.D., Stegman, E.C. & Gregor, B.L. Field comparison of irrigation scheduling methods for corn. *Transactions of the Asae* **37**, 1197-1203 (1994).
437.V., E.H. Irrigated corn (*zea mays*) yield response to nitrogen and water. (1984).
438.Musick, J.T. & Dusek, D.A. Irrigated corn yield response to water. *Transactions of the Asae* **23**, 92-98 (1980).
439.Vol., N. Irrigation scheduling with planned soil water depletion. *Transactions of the Asae* **37**, 1491-1497 (1994).
440.A, H.T., A, Y., D, S.A., A, D.D. & S, C.K. Yield and water use efficiency of corn in response to LEPA irrigation. **38**, 1737-1747 (1995).

441. Steiner, J.L., Howell, T.A., Tolk, J.A. & Schneider, A.D. in Irrigation and Drainage (1991) 297-3032015).
442. Payero, J.O., Klocke, N.L., Schneekloth, J.P. & Davison, D.R. Comparison of irrigation strategies for surface-irrigated corn in west central Nebraska. *Irrigation Sci* **24**, 257-265 (2006).
443. Payero, J.O., Melvin, S.R., Irmak, S. & Tarkalson, D. Yield response of corn to deficit irrigation in a semiarid climate. *Agr Water Manage* **84**, 101-112 (2006).
444. Mounce, R.B., O'Shaughnessy, S.A., Blaser, B.C., Colaizzi, P.D. & Evett, S.R. Crop response of drought-tolerant and conventional maize hybrids in a semiarid environment. *Irrigation Sci* **34**, 231-244 (2016).
445. Tolk, J.A., Howell, T.A. & Evett, S.R. Evapotranspiration and yield of corn grown on three high plains soils. *Agron J* **90**, 447-454 (1998).
446. Tolk, J.A., Howell, T.A. & Evett, S.R. Effect of mulch, irrigation, and soil type on water use and yield of maize. *Soil Till Res* **50**, 137-147 (1999).
447. Tolk, J.A., Evett, S.R., Xu, W.W. & Schwartz, R.C. Constraints on water use efficiency of drought tolerant maize grown in a semi-arid environment. *Field Crop Res* **186**, 66-77 (2016).
448. DeJonge, K.C., Andales, A.A., Ascough, J.C. & Hansen, N.C. Modeling of full and limited irrigation scenarios for corn in a semiarid environment. *T Asabe* **54**, 481-492 (2011).
449. Irmak, S. et al. Large-scale on-farm implementation of soil moisture-based irrigation management strategies for increasing maize water productivity. *T Asabe* **55**, 881-894 (2012).
450. Djaman, K. & Irmak, S. Soil water extraction patterns and crop, irrigation, and evapotranspiration water use efficiency of maize under full and limited irrigation and rained settings. *T Asabe* **55**, 1223-1238 (2012).
451. Dragicevic, V. et al. The effect of super-hydro-grow polymer on soil moisture, nitrogen status and maize growth. *Fresen Environ Bull* **20**, 1013-1019 (2011).
452. Hernandez, M. et al. Maize water use efficiency and evapotranspiration response to n supply under contrasting soil water availability. *Field Crop Res* **178**, 8-15 (2015).
453. Bushong, J.T., Miller, E.C., Mullock, J.L., Arnall, D.B. & Raun, W.R. Irrigated and rain-fed maize response to different nitrogen fertilizer application methods. *J Plant Nutr* **39**, 1874-1890 (2016).
454. Rudnick, D. et al. Economic return versus crop water productivity of maize for various nitrogen rates under full irrigation, limited irrigation, and rainfed settings in south central Nebraska. *J Irrig Drain Eng* **142** (2016).
455. Bennie, A.T.P. & Botha, F.J.P. Effect of deep tillage and controlled traffic on root-growth, water-use efficiency and yield of irrigated maize and wheat. *Soil Till Res* **7**, 85-95 (1986).
456. Rudnick, D.R. & Irmak, S. Impact of water and nitrogen management strategies on maize yield and water productivity indices under linear-move sprinkler irrigation. *T Asabe* **56**, 1769-1783 (2013).
457. Irmak, S. Interannual variation in long-term center pivot-irrigated maize

evapotranspiration and various water productivity response indices. I: grain yield, actual and basal evapotranspiration, irrigation-yield production functions, evapotranspiration-yield production functions, and yield response factors. *J Irrig Drain Eng* **141** (2015).
458. Howell, T.A., Copeland, K.S., Schneider, A.D. & Dusek, D.A. Sprinkler irrigation management for corn--southern Great Plains. *Transactions of the Asae* **32**, 147-154 (1989).
459. Howell, T.A., Schneider, A.D. & Evett, S.R. Subsurface and surface microirrigation of corn - - southern high plains. *Transactions of the Asae* **38**, 745-759 (1997).
460. Lamn, F.R., Manges, H.L., Stone, L.R., Khan, A.H. & Rogers, D.H. Water requirement of subsurface drip-irrigated corn in northwest Kansas. *Transactions of the Asae* **38**, 441-448 (1995).
461. Norwood, C.A. Water use and yield of limited-irrigated and dryland corn. *Soil Sci Soc Am J* **64**, 365-370 (2000).
462. Howell, T.A., Evett, S.R., Tolk, J.A., Schneider, A.D. & Steiner, J.L. Evapotranspiration of corn -- southern high plains. *Industrial Crops & Products* **34**, 1418-1422 (1996).
463. Evett, S.R., Howell, T.A., Schneider, A.D., Upchurch, D.R. & Wanjura, D.F. Canopy temperature based automatic irrigation control. (1996).
464. Hamilton, S.K., Hussain, M.Z., Bhardwaj, A.K., Basso, B. & Robertson, G.P. Comparative water use by maize, perennial crops, restored prairie, and poplar trees in the US Midwest. *Environ Res Lett* **10** (2015).
465. Lamm, F.R., Aiken, R.M. & Kheira, A.A.A. Corn yield and water use characteristics as affected by tillage, plant density, and irrigation. *T Asabe* **52**, 133-143 (2009).
466. Payero, J.O., Tarkalson, D.D., Irmak, S., Davison, D. & Petersen, J.L. Effect of timing of a deficit-irrigation allocation on corn evapotranspiration, yield, water use efficiency and dry mass. *Agr Water Manage* **96**, 1387-1397 (2009).
467. Alkaisi, M.M. & Yin, X. Effects of nitrogen rate, irrigation rate, and plant population on corn yield and water use efficiency. *Agron J* **95**, 1475-1482 (2003).
468. Sigua, G.C., Stone, K.C., Bauer, P.J. & Szogi, A.S. Nitrate leaching, water-use efficiency and yield of corn with different irrigation and nitrogen management systems in coastal plains, USA. In Environmental Impact 159-170(2016).
469. Waite, J. Corn and forage sorghum yield and water use in western Kansas. (2016).
470. Hernández, M. et al. Maize water use efficiency and evapotranspiration response to n supply under contrasting soil water availability. *Field Crop Res* **178**, 8-15 (2015)
471. Rodrigues, G.C., Martins, J.D., Da Silva, F.G., Carlesso, R. & Pereira, L.S. Modelling economic impacts of deficit irrigated maize in Brazil with consideration of different rainfall regimes. *Biosyst Eng* **116**, 97-110 (2013).
472. Otegui, M.E., Andrade, F.H. & Suero, E.E. Growth, water use, and kernel abortion of maize subjected to drought at silking. *Field Crop Res* **40**, 87-94 (1995).
473. Barbieri, P. et al. Maize evapotranspiration and water-use efficiency in response to

row spacing. *Agron J* **104**, 939-944 (2012).
